# Phenylspirodrimane with Moderate Reversal Effect of Multidrug Resistance Isolated from the Deep-Sea Fungus *Stachybotrys* sp. 3A00409

**DOI:** 10.3390/molecules29071685

**Published:** 2024-04-08

**Authors:** Xinhua Ma, Min Wu, Zhenwei Chen, Fan Cao, Tianhua Zhong, Zhuhua Luo, Zongze Shao, Yonghong Zhang, Limin Chen, Zhiqiang Zhang

**Affiliations:** 1 Fujian Provincial Key Laboratory of Pharmacology of Natural Medicine, School of Pharmacy, Fujian Medical University, Fuzhou 350122, China; maxinhua@fjmu.edu.cn (X.M.); wumin@fjmu.edu.cn (M.W.); chenyi1314170@163.com (Z.C.); caofan0101@163.com (F.C.); zhangyh@fjmu.edu.cn (Y.Z.); 2 Fuzhou Second Hospital, Fuzhou 350122, China; 3 Key Laboratory of Marine Biogenetic Resources, Third Institute of Oceanography, Ministry of Natural Sources, Xiamen 361005, China; zhongtianhua@tio.org.cn (T.Z.); luozhuhua@tio.org.cn (Z.L.); shaozongze@tio.org.cn (Z.S.)

**Keywords:** deep-sea fungus, *Stachybotrys*, phenylspirodrimanes, multidrug resistance (MDR), reversal MDR effects

## Abstract

Two new phenylspirodrimanes, stachybotrins K and L (**1** and **2**), together with eight known analogues (**3**–**10**), were isolated from deep-sea-derived *Stachybotrys* sp. MCCC 3A00409. Their structures were determined by extensive NMR data and mass spectroscopic analysis. Absolute configurations of new compounds were determined through a comparison of their circular dichroism (CD) spectra with other reported compounds. The possible reversal effects of all compounds were assayed in the resistant cancer cell lines. Stachybotrysin B (**8**) can reverse multidrug resistance (MDR) in ABCB1-overexpression cells (KBv200, Hela/VCR) at the non-cytotoxic concentration. Doxorubicin accumulation assay and molecular-docking analysis reveal that the mechanism of its reversal MDR effect may be related to the increase in the intracellular concentration of substrate anticancer drugs.

## 1. Introduction

Chemotherapy is the main treatment method for many malignant tumors. However, drug resistance is becoming a major problem in several cancers. Multidrug resistance (MDR) refers to the resistance of tumor cells to multiple chemotherapy agents, mediated by drug inactivation or the removal of drugs from target tumor cells [1,2]. MDR is currently considered to be one of the most common causes of cancer chemotherapy failure [3]. The most common mechanism of MDR is the overexpression of ABC transporters, which actively pump out large amounts of chemotherapy drugs from cancer cells, thereby weakening the efficacy of chemotherapy drugs [4,5]. The subfamily ABCB1/MDR1/P-glycoprotein, ABCC1/MRP1, and ABCG2/BCRP are the most extensively studied and considered as prime factors for the induction of MDR in tumor cells [6,7]. Among them, ABCB1/P-glycoprotein (P-gp) remains the best-studied and most potent ABC transporter to induce chemoresistance, and the upregulation of ABCB1/P-gp is closely linked with the emergence of MDR in tumor cells [8,9]. The discovery of ABCB1/P-gp suggests that combining ABCB1/P-gp inhibitors with traditional anticancer drugs may be a promising strategy for surmounting P-gp-mediated MDR [10,11]. In recent decades, three generations of ABCB1/P-gp inhibitors have been developed, some of which are currently undergoing clinical trials to evaluate their role in circumventing anti-cancer resistance [9,12,13,14,15,16] (see Table 1). However, due to their unacceptable toxicity and problematic pharmacokinetic interactions, many of these inhibitors have not been used in clinical practice [8,17,18]. Therefore, there is still a strong demand for the screening of multidrug-resistance reversal agents and the development of new multidrug-resistance antagonists.

Stachybotrys, a genus of filamentous fungi, is known to produce a broad range of secondary metabolites, such as macrocyclic trichothecenes, atranones, and phenylspirodrimanes (PSDs) [19,20]. PSDs belong to a type of meroterpenoids, which are proposed to be derived from the hybrid polyketide–terpenoid biosynthetic pathway. They feature a common drimane-type sesquiterpene skeleton connected with a benzene ring through a spirofuran ring and show high structural diversity [19]. To date, over 120 compounds of this class, including monomeric and dimeric PSDs, have been discovered to derive from Stachybotrys [19,20,21,22,23]. These metabolites are designated as the most dominant group of mycotoxins in this genus [19,20,24]. PSDs can be further divided into three main classes: tetracyclic aromatic sesquiterpenoids with alcohol and/or aldehyde side chains, like stachybotrydial, pentacyclic aromatic sesquiterpenoids, such as stachybotrylactams and stachybotrylactones, and stachyflins with pentacyclic moiety including a *cis*-fused decalin [22,25]. They display diverse biological activities, including anticomplement, antiviral (HIV-1 and IAV) and anti-inflammatory activity, cytotoxicity, neuroprotective effects, tyrosine kinase inhibition, and tumor-related kinases inhibition [19]. Active monomeric compounds are mostly concentrated in the first class with alcohol and/or aldehyde functionalities and stachybotrylactones [19].

**Figure 1:**
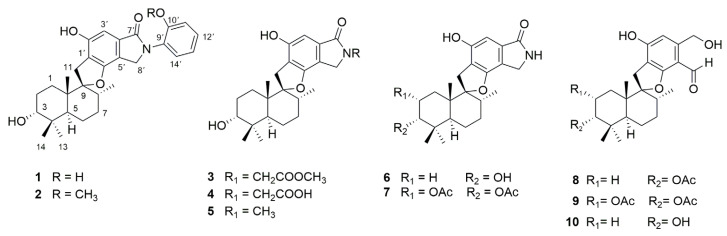
Chemical structures of compounds **1**–**10**.

In our prior work, a series of phenylspirodrimanes were isolated from a deep-sea-derived *Stachybotrys* sp. MCCC 3A00409 [26]. Attracted by their interesting structures, subsequent chemical investigation led to the isolation of two new phenylspirodrimanes (**1**–**2**), together with eight known derivatives (**3**–**10**), which were identified as stachybonoid E (**3**) [27], stachybotrin H (**4**) [26], stachybotrin E (**5**) [28], stachybotrylactam acetate (**6**) [29], α-acetoxystachybotrylactam acetate (**7**) [29], stachybotrysin B (**8**) [30], stachybotrysin H (**9**) [26], and Mer-NE5003E (**10**) [31] from the oats solid culture (Figure 1). In our research, we discovered that stachybotrysin B (**8**) had a reversal effect on MDR in ABCB1-overexpressing cancer cells. This paper described the isolation, structure elucidation, and bioactivity of these compounds.

## 2. Results and Discussion

### 2.1. Structural Determination

*Stachybotrys* sp. MCCC 3A00409 was cultured on solid oats and rice medium for 30 days at 28 °C. The organic extract prepared by solvent extraction was fractionated by repeated silica gel column chromatography (CC), ODS CC, Sephadex LH-20 CC, and, finally, semi-preparative HPLC to yield compounds **1**–**10** (Figure 1).

**Figure 2:**
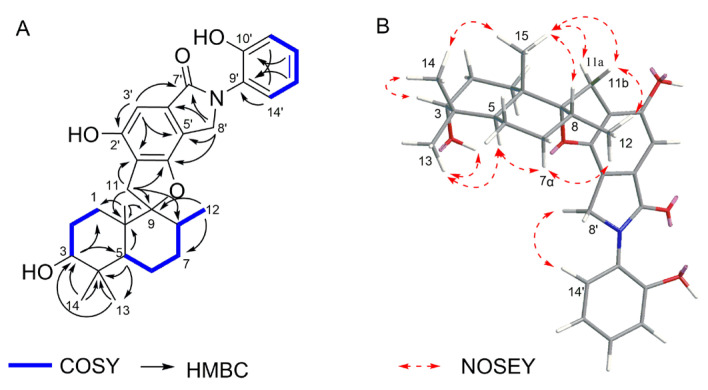
Key ^1^H-^1^H COSY, HMBC correlations (**A**) and NOESY correlations (**B**) of compound **1**.

**Figure 3:**
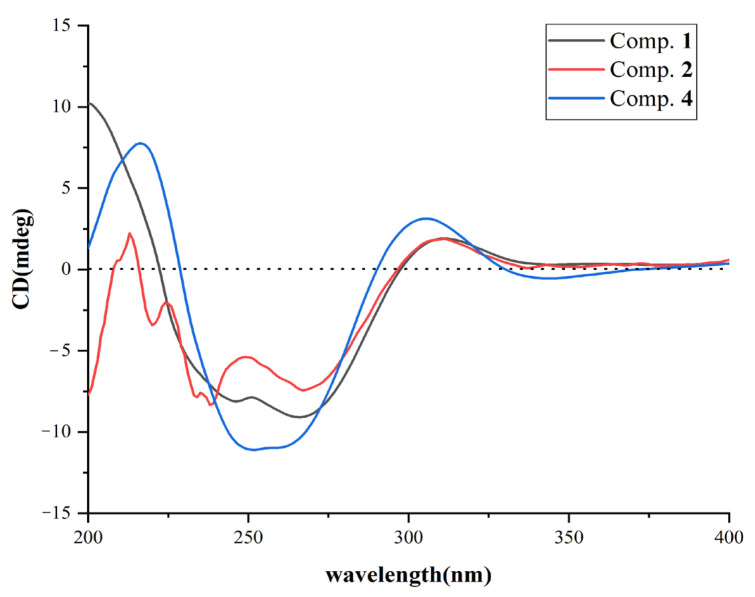
ECD spectra of compounds **1**, **2**, **4**.

Stachybotrin K (**1**) was obtained as a pale yellow oil. The HRESIMS peaks at *m*/*z* 478.2593 [M + H]^+^ suggested the molecular formula of C_29_H_35_NO_5_, requiring thirteen degrees of unsaturation. A comparison of the 1D NMR data (Table 2) with those of the known compound **5** (stachybotrin E) [28] revealed that they shared the same phenylspirodrimane skeleton, except for the replacement of N-CH_3_ in **5** by an ortho-substituted phenol group in **1**. The phenol unit was determined by the COSY correlations of H-11′/H-12′/H-13′/H-14′ and the HMBC correlations from H-11′ (*δ*_H_ 6.96, d, *J* = 8.0 Hz) to C-9′ (*δ*_C_ 125.7), C-10′ (*δ*_C_ 152.8), and C-13′ (*δ*_C_ 119.2), and from H-14′ (*δ*_H_ 7.31, d, *J* = 6.9 Hz) to C-9′, C-10′, C-12′ (*δ*_C_ 128.2) (Figure 2). Although the HMBC spectrum did not give the correlations from H-14′ to C-8′, the NOESY cross-peaks between H-14′ (*δ*_H_ 7.31) and H-8′ (*δ*_H_ 4.62), as shown in Figure 2, suggested that the phenol moiety was connected to the nitrogen atom. The planar structure of **1** was further confirmed by 2D NMR data (Figure 2). NOESY correlations H-3/Me-14, Me-14/Me-15, Me-15/H-8, Me-15/H_2_-11, and OH-3/Me-13, Me-13/H-5, H-5/H*α*-7, H*α*-7/Me-12 determined that H-3, Me-14, and Me-15 are in β-orientations, while Me-13, H-5, and Me-12 are in *α*-orientations (Figure 2). The absolute configuration of **1** was assigned to be 3*R*, 5*S*, 8*R*, 9*R*, and 10*S* on the basis of its ECD spectrum, which exhibited similar Cotton effects (CEs) (negative CEs were observed at 245 and 266 nm, while positive CEs were observed at 310 nm) (Figure 3) to those of stachybotrin E (**5**) [28].

Compound **2** was obtained as a pale yellow oil and was assigned the molecular formula of C_30_H_37_NO_5_ on the basis of HRESIMS at *m*/*z* 514.2570 [M + Na]^+^ (calcd. For 514.2569), along with ^1^H and ^13^C NMR spectroscopic data (Table 2). The structure of **2** was determined as a methylated analogue of **1** on the basis of the close similarity of NMR data, except for the presence of the methoxyl resonances at *δ*_H_ 3.79 (3H, s) and *δ*_C_ 55.6, while the methoxy group was substituted at OH-10′, as evident from the correlation between H_3_-15′ (*δ*_H_ 3.79) and C-10′ (*δ*_C_ 154.9) (Supplementary Materials Figure S1). The relative configuration of the phenylspirodrimane moiety in **2** was further established by its NOESY correlations (Figure S1). According to the similar chemical shifts from **2** to **1** (Figure 3) and the associated Cotton effects (CEs), the absolute configuration of compound **2** was identified as being as same as **1**. Therefore, the structure of compound **2** was determined and given the name of stachybotrin L. When compound **1** was stirred with silica in MeOH for 48 h, compound **2** was not detected by HPLC-UV analysis. Therefore, we confirmed that compound **2** was not an artifact product.

Stachybotrin H (**4**) was previously isolated from the same strain [26], but the absolute configuration was not established. Through a comparison of its ECD curves with **1** and **2** (Figure 3), the absolute configuration of **4** was also established as 3*R*, 5*S*, 8*R*, 9*R*, and 10*S*.

### 2.2. Biological Assays

**Figure 4:**
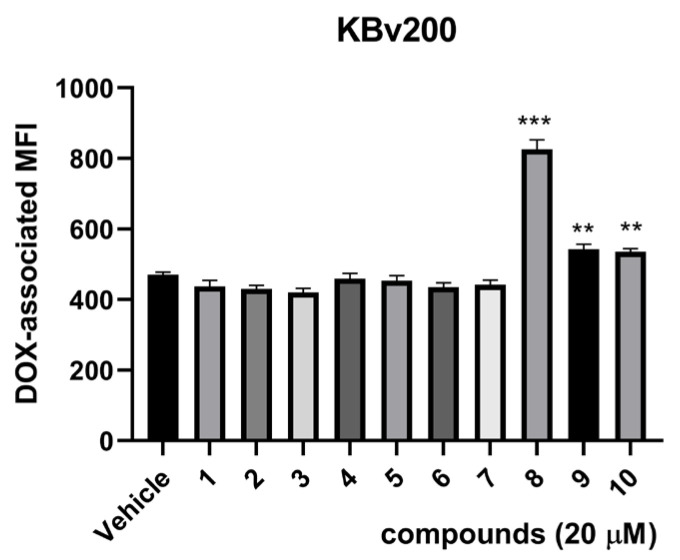
The effect of compounds **1**–**10** on the accumulation of DOX in KBv200 cells. ** *p* < 0.01, *** *p* < 0.001 versus control group.

To quickly screen for compounds with resistance reversal activity, we used flow cytometry analysis to detect the effects of compounds **1**–**10** on the accumulation of doxorubicin (DOX) in the resistant human oral epithelial cancer cell line KBv200. The results showed that compound **8** could increase the accumulation of DOX in KBv200 cells at 20 μM (Figure 4).

**Figure 5:**
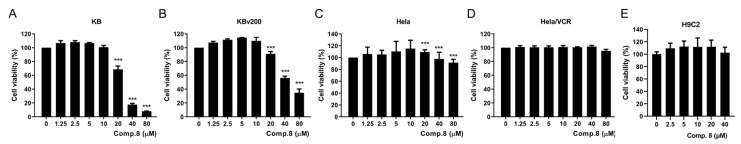
Cytotoxicity of compound **8** (comp. **8**) in pairs of cancer cells or normal cells. (**A**) ABCB1-negative KB cells; (**B**) ABCB1-overexpressing KBv200 cells; (**C**) ABCB1-negative Hela cells; (**D**) ABCB1-overexpressing Hela/VCR cells; (**E**) rat cardiomyocytes H9C2 cells. *** *p* < 0.001 versus control group.

To find the suitable concentrations of compound **8** for reversing MDR in vitro, we first examined the cytotoxic effect of **8** on different cancer cell lines via MTT assay. As shown in Figure 5, stachybotrysin B (**8**) had almost no significant cytotoxic effect on KB, KBv200 cells at concentrations below 10 μM, and had no cytotoxic effects on Hela and Hela/VCR below 80 μM, after 72 h of treatment. In addition, compound 8 showed no cytotoxic activity against normal H9C2 cells below 40 μM (Figure 5E). Thus, we chose concentrations of 10 μM and 20 μM as the maximum concentrations for further reversal assays in KBv200 and Hela/VCR cell lines, respectively; at the chosen concentrations, more than 90% of cells survived.

Then, we investigated the reversal effect of compound **8** on different tumor-sensitive cell lines and their drug-resistant cell lines. The results (Table 3) revealed that compound **8** exhibited moderate resistance reversal effects against the substrate chemotherapeutic agents, DOX and navelbine (NVB), in the ABCB1-overexpressing drug-resistant cell line KBv200, and against DOX in the ABCB1-overexpressing drug-resistant cell line Hela/VCR, with fold resistance ranging from 1.54 to 4.86 and from 1.43 to 7.82, respectively, in a dose-dependent manner. However, compound **8** did not significantly decrease the IC_50_ of cisplatin, which was not a substrate chemotherapeutic drug of ABCB1, in the ABCB1-overexpressing MDR cells. These results indicated that compound **8** could reverse ABCB1-mediated MDR.

**Figure 6:**
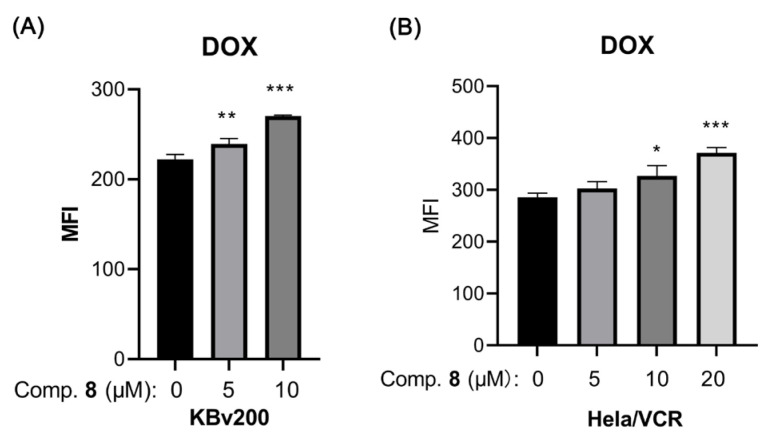
Effect of compound **8** on the accumulation of doxorubicin (DOX) in ABCB1-overexpressing KBv200 (**A**) and Hela/VCR (**B**) cells. The results are presented as the mean fluorescence intensity (MFI) in ABCB1-overexpressing cells. * *p* < 0.05, ** *p* < 0.01, *** *p* < 0.001 versus the control group.

We further detected an effect of compound **8** on the intracellular accumulation of DOX, the substrate agents of the ABCB1 transporter, in ABCB1-overexpressing cell lines (KBv200, Hela/VCR) via flow cytometry analysis. The results showed that compound **8** could increase the accumulation of DOX in KBv200 and Hela/VCR, thereby increasing the concentration of DOX in these cancer cell lines at concentrations below 10 μM and 20 μM, respectively (Figure 6).

### 2.3. Docking Analysis of Compound 8 and Verapamil with ABCB1 

**Figure 7:**
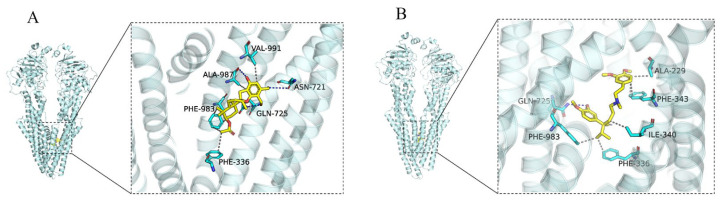
Docking of compound **8** (**A**) and verapamil (**B**) in the drug-binding pocket of ABCB1. Compound **8** and verapamil are shown as molecular models via yellow sticks. The light cyan cartoon represents ABCB1. Blue dashed lines represent hydrogen bonding, and gray dashed lines represent hydrophobic interactions.

Molecule docking simulation technology is a convenient and effective method to explore the interaction between small molecules and targets [32,33,34,35]. We performed a molecular docking simulation to characterize the molecular basis of the interactions between compound **8** and the human homology model of the ABCB1 transporter protein structure (PDB ID: 6QEX). The results (Figure 7) revealed that **8** was well-fitted at the substrate-binding site in the transmembrane domains (TMDs), with the highest docking score of −7.4 kcal/mol. Then, we chose this protomol to dock verapamil (a classical ABCB1/P-gp inhibitor) [9] to TMDs. The docking score was −7.7 kcal/mol.

For verapamil, a hydrogen bond is formed between the hydroxy group and GLN725 of ABCB1, and hydrophobic interactions are formed between verapamil and five amino acids, like ILE-340, PHE-983, PHE-336, ALA-229, and PHE-343 (Figure 7B). Similar to verapamil, compound **8** also interact with Gln725 of ABCB1 through H-bonds (Figure 7A). In addition, a hydrogen bond was found between the hydroxyl and aldehyde groups at the aromatic ring of and ALA-987 and ASN-721. The molecule was also predicted to interact with PHE-983, ALA-987, VAL-991, and PHE-336 via hydrophobic interaction. Thus, we can infer that the ABCB1 protein might recognize both **8** and verapamil through a similar mechanism.

## 3. Materials and Methods

### 3.1. General Experimental Procedure

Optical rotations were performed using a JASCO P-1020 digital polarimeter (Jasco Corporation, Tokyo, Japan). ECD spectra were obtained with a JASCO J-815 spectropolarimeter (Jasco Corporation, Tokyo, Japan). UV spectra were obtained using a Shimadz UV-210A spectrometer (Shimadzu Corporation, Tokyo, Japan). NMR spectra were recorded with 400 and 600 MHz Bruker Avance NMR spectrometers (Bruker BioSpin AG, Fällanden, Switzerland). ESIMS was performed using a Shimadzu LCMS-8040 Liquid Chromatograph Mass Spectrometer (Shimadzu Corporation, Tokyo, Japan). HRESIMS spectra were obtained using a Thermo Scientific Ultimate 3000 UHPLC-Q Exactive spectrometer (ThermoFisher Scientific, Waltham, MA, USA). Semi-preparative HPLC was performed on a Waters 1525 system using a semi-preparative C18 (Waters SunFire C18 ODB Prep Column, 10 × 250 mm, 5 µm, 3 mL/min) column coupled with a Waters 2996 photodiode array detector (Waters Corporation, Milford, MA, USA). Thin-layer chromatography (TLC) was performed on plates precoated with silica gel GF254 (10–40 μm) (Qingdao Marine Chemical Factory, Qingdao, China). Sephadex LH-20 (Amersham Biosciences, Uppsala, Sweden) and silica gel (100–200 and 200–300 mesh, Qingdao Marine Chemical Factory) were used for column chromatography.

### 3.2. Fungal Material

The fungus strain *Stachybotrys* sp. MCCC 3A00409 was isolated from Atlantic Ocean (−2807 m), as described previously [26]. The strain was deposited in the Marine Culture Collection Center (MCCC), Third Institute of Oceanography, Ministry of Natural Sources, and School of Pharmacy, Fujian Medical University.

The fungal strain was inoculated in the liquid medium (2.0% mannitol, 2.0% maltose, 1.0% glucose, 1.0% monosodium glutamate, 0.05% KH_2_PO_4_, 0.03% MgSO_4_·7H_2_O, 0.3% yeast extract, 0.1% corn steep liquor, and 3.0% sea salt) in 500 mL shake flask with a loading of 150 mL as seed culture and incubated on a rotary shaker (165 rpm) at 28 °C for 2 days. A massive fermentation occurred in the solid medium (70 g oats, 20 g rice, distilled H_2_O 150 mL) using 300 mL × 60 Erlenmeyer flasks (1 L) and shaker fermentation at 28 °C for 35 days.

### 3.3. Extraction and Isolation

After incubation, the solid culture was extracted with CH_3_OH three times. The CH_3_OH extract was evaporated under reduced pressure to obtain an aqueous solution, and then the aqueous solution was extracted three times with EtOAc to obtain a brown crude gum (30.2 g).

The crude EtOAc extract was applied to a silica gel (200–300 mesh) column (180 g, 6.0 × 16.5 cm) and was separated into six fractions (Frs.1–6) using a step gradient elution of petroleum ether/CH_2_Cl_2_ and CH_2_Cl_2_/CH_3_OH. Fr.3 (3.6 g) was separated on a C-18 ODS column with a CH_3_OH-H_2_O gradient (5–100%), producing six subfractions (Fr.3.1–Fr.3.6). Fr.3.3 was separated by Sephadex LH-20 using CH_3_OH as the eluting solvent, and then on a semi-preparative HPLC column (75% CH_3_OH/H_2_O, 3.0 mL/min) to afford compounds **6** (20 mg, *t*_R_ = 32 min), **5** (5 mg, *t*_R_ = 35 min), and **7** (14 mg, *t*_R_ = 43 min). Fr.4 (5.1 g) was separated on a C-18 ODS column using step-gradient elution with CH_3_OH/H_2_O (5–100%) to obtain six subfractions (Fr.4.1–Fr.4.6). Subfraction Fr.4.3 was separated into seven subfractions (Fr.4.3.1–Fr.4.3.7) by Sephadex LH-20 eluted with CH_3_OH. Fr.4.3.3 was purified by semi-preparative HPLC (75% CH_3_OH/H_2_O, 3.0 mL/min) to obtain compounds **3** (12 mg, *t*_R_ = 26 min) and **4** (8 mg, *t*_R_ = 31 min). Fr.4.3.4 was purified by semi-preparative HPLC (75% CH_3_OH/H_2_O, 3.0 mL/min) to obtain compounds **10** (7 mg, *t*_R_ = 30 min), **8** (20 mg, *t*_R_ = 34 min), and **9** (11 mg, *t*_R_ = 40 min). Fr.5 (2.4 g) was fractionated by Sephadex LH-20 eluted with CH_3_OH and was further subjected, via semi-preparative HPLC (55% CH_3_CN/H_2_O, 3.0 mL/min) to obtain compounds **1** (6 mg, *t*_R_ = 36 min) and **2** (5 mg, *t*_R_ = 40 min).

Stachybotrin K (**1**): pale yellow oil; [*α*]^24^_D_ −35.4 (*c* 0.10, MeOH); UV (MeOH) *λ*_max_ (log *ε*) 217 (3.93), 264 (3.05), 305 (2.55) nm; ECD (1.45 × 10^−4^ M, MeOH) λmax (Δε): 200 (+10.24), 246 (−8.12), 266 (−9.08), and 311 (+1.90) nm; ^1^H and ^13^C NMR data shown in Table 1; HRESIMS: [M + H]^+^*m*/*z* 478.2593 (calcd. for C_29_H_36_NO_5_, 478.2593).

Stachybotrin L (**2**): pale yellow oil; [*α*]^24^_D_ −38.2 (*c* 0.10, MeOH); UV (MeOH) *λ*_max_ (log *ε*) 216 (4.02), 263 (3.16), 303 (2.68) nm; ECD (1.05 × 10^−4^ M, MeOH) λmax (Δε): 213 (+2.22), 238 (−8.32), 267 (−7.43), and 311 (+1.87) nm; ^1^H and ^13^C NMR data shown in Table 1; HRESIMS: [M + Na]^+^*m*/*z* 514.2570 (calcd. for C_30_H_37_NO_5_Na, 514.2569).

Stachybotrin H (4): colorless oil; ECD (1.33 × 10^−4^ M, MeOH) λmax (Δε): 216 (+7.76), 252 (−11.10), 305 (+3.12), and 344 (−0.55) nm.

### 3.4. Cells and Materials

KB (human oral epidermoid carcinoma cell line) and its vincristine-selected ABCB1-overexpressing cell line KBv200, Hela (human cervical carcinoma cell line) and its vincristine-selected ABCB1-overexpressing cell line Hela/VCR, and rat cardiomyocytes cells H9C2 were cultured in RPMI 1640 medium supplemented with 10% FBS in the presence of 5% CO_2_ at 37 °C. The commercial cell lines shown above were originally obtained from American-type culture collection (ATCC). All cells were grown in drug-free culture medium for more than 2 weeks before assay. Doxorubicin (DOX), navelbine (NVB), cisplatin, 3-(4, 5-dimethylthiazol-2-yl)-2, 5-diphenyltetrazolium bromide (MTT), and dimethyl sulfoxide (DMSO) were all purchased from Sigma-Aldrich (St. Louis, MO, USA).

### 3.5. Cell Cytotoxicity Assay

Cell cytotoxicity assay in vitro was evaluated by MTT assay, as described previously [36]. In brief, cells were seeded in 96-well plates at an appropriate density and incubated for 24 h at 37 °C. Then, the cells were treated with various concentrations of chemotherapeutic durgs or compounds. A total of 20 μL of MTT solution (5 mg/mL in PBS) was added to each well after 72 h incubation. The plates were incubated at 37 °C for another 4 h and the resulting formazan crystals were dissolved with 150 μL DMSO. Absorbance in each well was measured by Model 550 Microplate Reader (Bio-Rad, Hercules, CA, USA) at 570 nm. The half maximal (50%) inhibitory concentration (IC_50_) was calculated from the curves using GraphPad Prism 9.0, using the Bliss method [37]. The maximal concentration of compound **8** indicated that a cell survival of more than 80% was required when testing its MDR-reversal effect. The effect of the MDR reversal by compound **8** was calculated by dividing the IC_50_ of cells treated with chemotherapeutic drugs alone or by the IC_50_ of cells treated with chemotherapeutic drugs in the presence of compound **8**. All experiments were repeated at least three times.

### 3.6. DOX Accumulation Assay

A flow cytometry assay was performed to examine whether compounds **1**–**10** could affect the accumulation of DOX in KBv200 or Hela/VCR cells, as previously described [36]. In brief, the logarithmically growing KBv200 or Hela/VCR cells were seeded in six-well plates at a density of 3 × 10^5^ per well. After 24 h of incubation, the cells were pretreated with 20 μM of compounds **1**–**10** or various concentrations of compound **8** or the vehicle for 3 h at 37 °C. Then, cells were further incubated with 10 μM DOX for another 3 h. After that, the cells were collected, washed three times with cold phosphate-buffered saline (PBS), and resuspended in 500 μL PBS. The mean fluorescence intensity (MFI) of DOX was examined via flow cytometry (Cytomics FC500, Beckman Coulter Inc., Brea, CA, USA) with an excitation wavelength of 488 nm and an emission wavelength of 550 nm.

### 3.7. Molecular Docking Analysis

The crystal structures of the ABCB1 protein [38] (PDB ID: 6QEX) used for docking were downloaded from the PDB database. The 3D structure of the small molecule complex was constructed using Chem3D 20.0 and energy was minimized under the MMFF94 force field. Before ligand docking, PyMol 2.5.5 [39] was used to treat the receptor protein, including the removal water molecules, salt ions, and small molecules. Subsequently, a 25 × 25 × 25 grid box, with the center being the center of the crystal ligand, was set up. In addition, ADFRsuite 1.0 [40] was used to convert all processed small molecules and receptor proteins into the necessary PDBQT format for AutoDock Vina 1.1.2 [41] docking software. When docking, the global search granularity was set to 32, while the remaining parameters remained at their default settings. The top-scoring model (based on the docking score in kcal/mol) was visualized and analyzed using PyMol 2.5.5.

### 3.8. Data Analysis

All data are expressed as mean ± standard deviation (mean ± SD) established in different experiments and analyzed by one-way analysis of variance (ANOVA), followed by Bonferroni post hoc test. Statistical graphs were produced by GraphPad Prism 9.0.0 software (GraphPad Software, Inc., San Diego, CA, USA). *, *p* < 0.05; **, *p* < 0.01; ***, *p* < 0.001, was considered statistically significant.

## 4. Conclusions

In this study, two new phenylspirodrimanes, stachybotrin K (**1**), and stachybotrin L (**2**), together with eight known derivatives (**3**–**10**), were isolated from deep-sea-derived *Stachybotrys* sp. MCCC 3A00409. Extensive NMR spectroscopic analysis and ECD analysis were used to elucidate the structures of new compounds, including their absolute configurations. The screening results of all compounds with resistance reversal activity in a drug-resistant tumor cell line showed that stachybotrysin B (**8**) had the most significant effect. Compound **8** exhibited resistance reversal effects against the substrate chemotherapeutic agents, doxorubicin (DOX), and navelbine (NVB) in the ABCB1-overexpressing drug-resistant cell line KBv200, and against DOX in the ABCB1-overexpressing drug-resistant cell line Hela/VCR, in a dose-dependent manner. The results of flow cytometry analysis showed that the mechanism of its MDR reversal effect might be related to the increase in the intracellular concentration of substrate anticancer drug. The docking analysis of compound **8** binding to ABCB1 transporter protein indicated that **8** can potentially strongly interact with several amino acid residues within the transmembrane regions of ABCB1. This interaction might inhibit the efflux of substrates such as DOX, thereby increasing the accumulation of DOX in ABCB1-resistant cancer cells and reversing ABCB1-mediated multidrug resistance.

## Figures and Tables

**Table 1:** Summary of major ABCB1/P-gp inhibitors in clinical development.

Name	Chemical Structure	Mechanism of Action	Adverse Effects	Phase
First generation				
Verapamil	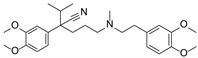	Calcium channel blocker	Hypotension, cardiotoxicity	Phase II
Cyclosporine A	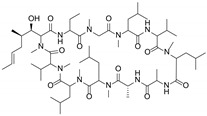	P-gp inhibitor	Not significant	Phase II
Qunine	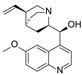	P-gp inhibitor	Myelosuppression	Phase III
Second generation				
Dexverapamil	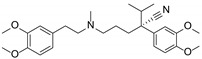	Calcium channel blocker	Cardiotoxicity	Phase II
Valspodar (PSC833)	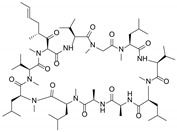	P-gp inhibitor	Not significant	Phase III
Biricodar (VX-710)	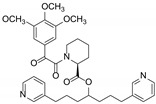	P-gp inhibitor	Neutropenia	Phase II
Third generation				
Laniquidar (R101933)	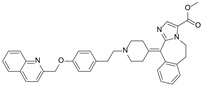	P-gp inhibitor	Mucositis and neutropenic fever	Phase II
Zosuquidar (LY335979)	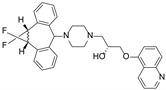	P-gp inhibitor	Neurotoxicity	Phase III
Tariquidar (XR9576)	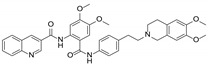	P-gp inhibitor	Not significant	Phase II
ONT-093	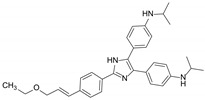	P-gp inhibitor	Not significant	Phase I

**Table 2:** 1D NMR data (400/100 MHz, DMSO-*d*_6_) for compounds **1** and **2**.

No.	1		2	
	*δ*_C_	*δ*_H_ (*J* in Hz)	*δ*_C_	*δ*_H_ (*J* in Hz)
1	23.9, CH_2_	0.94, m; 1.79, m	23.9, CH_2_	0.94, m; 1.78, m
2	24.9, CH_2_	1.37, m; 1.76, m	24.9, CH_2_	1.36, m; 1.75, m
3	73.5, CH	3.17, brs	73.5, CH	3.16, brs
4	36.5, C		36.5, C	
5	39.9, CH	2.01, d (8.3)	39.9, CH	2.00, m
6	20.5, CH_2_	1.42, m; 1.46, m	20.5, CH_2_	1.41, m; 1.45, m
7	30.8, CH_2_	1.39, m; 1.52, m	30.8, CH_2_	1.39, m; 1.51, m
8	37.3, CH	1.82, m	37.3, CH	1.81, m
9	98.1, C		98.1, C	
10	41.8, C		41.9, C	
11	31.7, CH_2_	3.14, d (17.5) 2.79 (d, 17.0)	31.7, CH_2_	3.14, d (17.9) 2.79, d (17.1)
12	15.6, CH_3_	0.68, d (6.4)	15.6, CH_3_	0.68, d (6.4)
13	28.7, CH_3_	0.86, s	28.7, CH_3_	0.86, s
14	22.4, CH_3_	0.79, s	22.4, CH_3_	0.79, s
15	15.8, CH_3_	0.95, s	15.8, CH_3_	0.95, s
1′	117.1, C		117.2 s	
2′	153.8, C		153.9 s	
3′	101.1, CH	6.64, s	101.2, CH	6.64, s
4′	133.6, C		133.5, C	
5′	112.5, C		112.4, C	
6′	155.9, C		155.9, C	
7′	167.2, C		167.1, C	
8′	48.8, CH_2_	4.62, brs	49.0, CH_2_	4.53, dd (16.2) 4.61, dd (16.2)
9′	125.7, C		126.8, C	
10′	152.8, C		154.9, C	
11′	116.8, CH	6.96, d (8.0)	112.4, CH	7.15, d (8.2)
12′	128.2, CH	7.17, t (7.6)	128.7, CH	7.34, t (7.8)
13′	119.2, CH	6.86, t (7.5)	120.5, CH	7.01, t (7.6)
14′	128.6, CH	7.31, d (6.9)	129.0, CH	7.36, dd (7.6, 1.6)
15′			55.6, CH_3_	3.79 s
3-OH		4.09, d (2.9)		4.11, d (2.6)

**Table 3:** MDR-reversal effects of compound **8** on ABCBl substrate drugs and non-substrate drugs.

Compound	IC_50_ ± SD μM (Fold Reversal)			
	KB		KBv200	
Doxorubicin	0.007 ± 0.008		3.706 ± 1.224	
+5 μmol L^−1^ **8**	0.007 ± 0.007	1.00	2.414 ± 1.814	1.54
+10 μmol L^−1^ **8**	0.007 ± 0.007	1.00	1.897 ± 1.648	1.95
Navelbine	0.001 ± 0.0001		2.514 ± 2.271	
+5 μmol L^−1^ **8**	0.001 ± 0.0001	1.00	0.800 ± 0.240	3.14
+10 μmol L^−1^ **8**	0.001 ± 0.0001	1.00	0.517 ± 0.007	4.86
Cisplatin	0.385 ± 0.204		0.810 ± 0.377	
+5 μmol L^−1^ **8**	0.455 ± 0.234	0.84	0.913 ± 0.426	0.89
+10 μmol L^−1^ **8**	0.493 ± 0.228	0.77	1.081 ± 0.482	0.75
	Hela		Hela/VCR	
Doxorubicin	0.098 ± 0.039		0.789 ± 0.575	
+5 μmol L^−1^ **8**	0.074 ± 0.029	1.32	0.553 ± 0.410	1.43
+10 μmol L^−1^ **8**	0.066 ± 0.018	1.48	0.324 ± 0.364	2.44
+20 μmol L^−1^ **8**	0.083 ± 0.010	1.18	0.101 ± 0.045	7.82
Cisplatin	1.812 ± 1.075		0.873 ± 0.047	
+5 μmol L^−1^ **8**	1.472 ± 0.861	1.23	0.954 ± 0.081	0.92
+10 μmol L^−1^ **8**	1.749 ± 0.956	1.04	1.032 ± 0.032	0.85
+20 μmol L^−1^ **8**	2.227 ± 1.289	0.81	1.363 ± 0.348	0.64

## Data Availability

Data are contained within the article and Supplementary Materials.

## Supplementary Materials

The following supporting information can be downloaded at: https://www.mdpi.com/article/10.3390/molecules29071685/s1, Figure S1: Key ^1^H-^1^H COSY, HMBC, and NOESY correlations of **2**; Figure S2: ^1^H NMR spectrum (400 MHz) of stachybotrin K (**1**) in DMSO-*d*_6_; Figure S3: ^13^C NMR spectrum (100 MHz) of stachybotrin K (**1**) in DMSO-*d*_6_; Figure S4: ^1^H NMR spectrum (600 MHz) of stachybotrin K (**1**) in DMSO-*d*_6_; Figure S5: ^13^C NMR spectrum (150 MHz) of stachybotrin K (**1**) in DMSO-*d*_6_; Figure S6: DEPT 90 spectrum (100 MHz) of stachybotrin K (**1**) in DMSO-*d*_6_; Figure S7: DEPT 135 spectrum (100 MHz) of stachybotrin K (**1**) in DMSO-*d*_6_; Figure S8: HMQC spectrum of stachybotrin K (**1**) in DMSO-*d*_6_; Figure S9: ^1^H-^1^H COSY spectrum of stachybotrin K (**1**) in DMSO-*d*_6_; Figure S10: HMBC spectrum of stachybotrin K (**1**) in DMSO-*d*_6_; Figure S11: ROSEY spectrum of stachybotrin K (**1**) in DMSO-*d*_6_; Figure S12: HRESIMS spectrum of stachybotrin K (**1**); Figure S13: ^1^H NMR spectrum (400 MHz) of stachybotrin L (**2**) in DMSO-*d*_6_; Figure S14: ^13^C NMR spectrum (100 MHz) of stachybotrin L (**2**) in DMSO-*d*_6_; Figure S15: ^1^H NMR spectrum (600 MHz) of stachybotrin L (**2**) in DMSO-*d*_6_; Figure S16: ^13^C NMR spectrum (150 MHz) of stachybotrin L (**2**) in DMSO-*d*_6_; Figure S17: DEPT 90 spectrum (100 MHz) of stachybotrin K (**2**) in DMSO-*d*_6_; Figure S18: DEPT 135 spectrum (100 MHz) of stachybotrin K (**2**) in DMSO-*d*_6_; Figure S19: HMQC spectrum of stachybotrin L (**2**) in DMSO-*d*_6_; Figure S20: ^1^H-^1^H COSY spectrum of stachybotrin L (**2**) in DMSO-*d*_6_; Figure S21: HMBC spectrum of stachybotrin L (**2**) in DMSO-*d*_6_; Figure S22: ROSEY spectrum of stachybotrin L (**2**) in DMSO-*d*_6_; Figure S23: HRESIMS spectrum of stachybotrin L (**2**).
