# Supplementary material for: Phenylspirodrimane with Moderate Reversal Effect of Multidrug Resistance Isolated from the Deep-Sea Fungus Stachybotrys sp. 3A00409
Source: Molecules. 2024 Apr 8;29(7):1685. doi: 10.3390/molecules29071685 (PMC11013241; doi:10.3390/molecules29071685)
Supplement: Supplementary file 1 [file molecules-29-01685-s001.zip › molecules-2923868-supplementary.pdf]

Supplementary Material

Phenylspirodrimane with Moderate Reversal Effect of Multidrug Resistance Isolated from The Deep-sea Fungus *Stachybotrys* sp. 3A00409

Xin-Hua Ma [1], Min Wu [1,2], Zhen-Wei Chen [1], Fan Cao [1], Tian-Hua Zhong[3], Zhu-Hua Luo [3], Zong-Ze Shao [3], Yong-Hong Zhang [1], Li-Min Chen [1,*] and Zhi-Qiang Zhang [1,*]

[1] Fujian Provincial Key Laboratory of Pharmacology of Natural Medicine, School of Pharmacy, Fujian Medical University, Fuzhou 350122, China
[2] Fuzhou Second Hospital, Fuzhou 350122, China
[3] Key Laboratory of Marine Biogenetic Resources, Third Institute of Oceanography, Ministry of Natural Sources, Xiamen, Fujian 361005, China
* Correspondence: Zhi-Qiang Zhang, zzq@fjmu.edu.cn; Li-Min Chen, chenlm5696@163.com

Abstract: Two new phenylspirodrimanes, stachybotrins K and L (**1** and **2**) together with eight known analogues (**3-10**) were isolated from deep-sea derived *Stachybotrys* sp. MCCC 3A00409. Their structures were determined by extensive NMR data and mass spectroscopic analysis. Absolute configurations of new compounds were determined by comparison of their circular dichroism (CD) spectra with other reported compounds. The possible reversal effects of all compounds were assayed the in the resistant cancer cell lines. Stachybotrysin B (**8**) can reverse multidrug resistance (MDR) in ABCB1 over expression cells (KBv200, Hela/VCR) at the non-cytotoxic concentration. Doxorubicin accumulation assay and molecular-docking analysis reveal that the mechanism of its reversal MDR effect may be related to the increase of the intracellular concentration of substrate anticancer drugs.

Keywords: deep-sea fungus; *Stachybotrys*; phenylspirodrimanes; multidrug resistance (MDR); reversal MDR effects

List of Supporting Information

Figure S1 Key ^{1}H-^{1}H COSY, HMBC and NOESY correlations of **2**

Figure S2 ^{1}H NMR Spectrum (400 MHz) of stachybotrin K (**1**) in DMSO-d_6

Figure S3 ^{13}C NMR Spectrum (100 MHz) of stachybotrin K (**1**) in DMSO-d_6

Figure S4 ^{1}H NMR Spectrum (600 MHz) of stachybotrin K (**1**) in DMSO-d_6

Figure S5. ^{13}C NMR Spectrum (150 MHz) of stachybotrin K (**1**) in DMSO-d_6

Figure S6 DEPT 90 Spectrum (100 MHz) of stachybotrin K (**1**) in DMSO-d_6

Figure S7 DEPT 135 Spectrum (100 MHz) of stachybotrin K (**1**) in DMSO-d_6

Figure S8 HMQC Spectrum of stachybotrin K (**1**) in DMSO-d_6

Figure S9 ^{1}H-^{1}H COSY Spectrum of stachybotrin K (**1**) in DMSO-d_6

Figure S10 HMBC Spectrum of stachybotrin K (**1**) in DMSO-d_6

Figure S11 ROSEY Spectrum of stachybotrin K (**1**) in DMSO-d_6

Figure S12 HRESIMS Spectrum of stachybotrin K (**1**)

Figure S13 ^{1}H NMR Spectrum (400 MHz) of stachybotrin L (**2**) in DMSO-d_6

Figure S14 ^{13}C NMR Spectrum (100 MHz) of stachybotrin L (**2**) in DMSO-d_6

Figure S15 ^{1}H NMR Spectrum (600 MHz) of stachybotrin L (**2**) in DMSO-d_6

Figure S16 ^{13}C NMR Spectrum (150 MHz) of stachybotrin L (**2**) in DMSO-d_6

Figure S17 DEPT 90 Spectrum (100 MHz) of stachybotrin L (**2**) in DMSO-d_6

Figure S18 DEPT 135 Spectrum (100 MHz) of stachybotrin L (**2**) in DMSO-d_6

Figure S19 HMQC Spectrum of stachybotrin L (**2**) in DMSO-d_6

Figure S20 ^{1}H-^{1}H COSY Spectrum of stachybotrin L (**2**) in DMSO-d_6

Figure S21 HMBC Spectrum of stachybotrin L (**2**) in DMSO-d_6

Figure S22 ROSEY Spectrum of stachybotrin L (**2**) in DMSO-d_6

Figure S23 HRESIMS Spectrum of stachybotrin L (**2**)

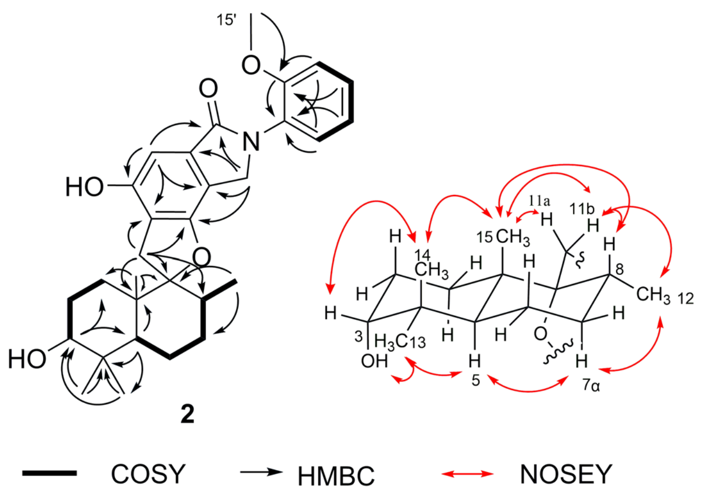

Figure S1 Key ^{1}H-^{1}H COSY, HMBC and NOESY correlations of compound **2**.

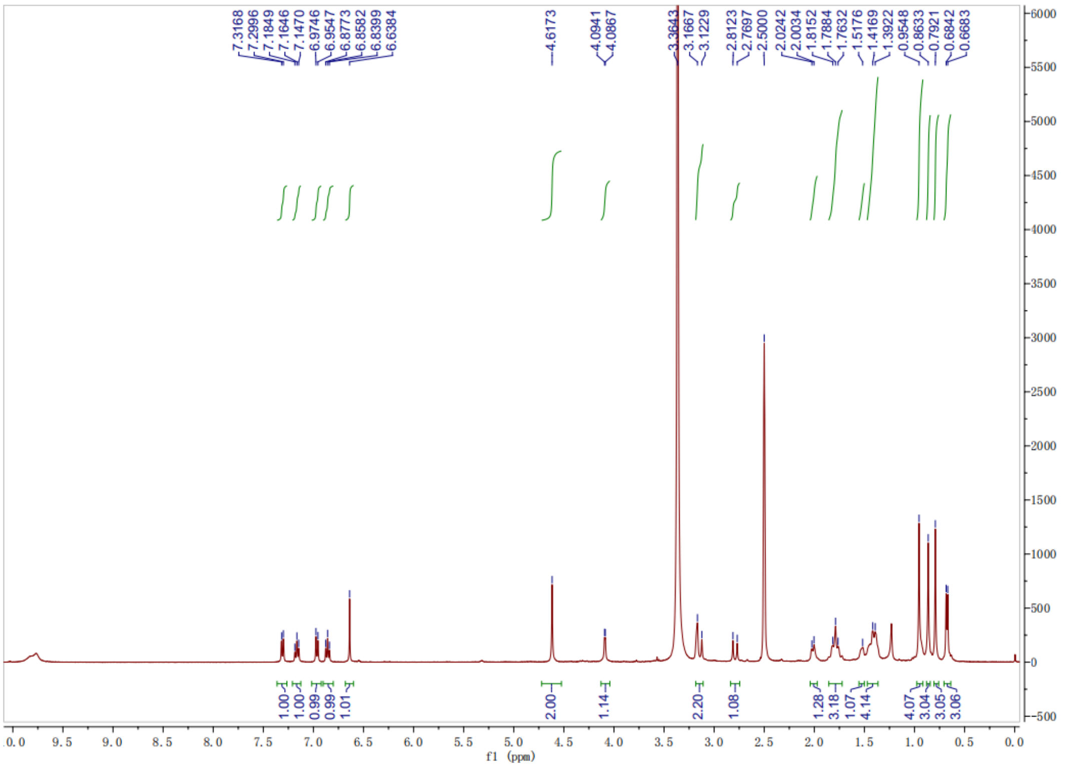

Figure S2 ^{1}H NMR Spectrum (400 MHz) of stachybotrin K (**1**) in DMSO-d_6

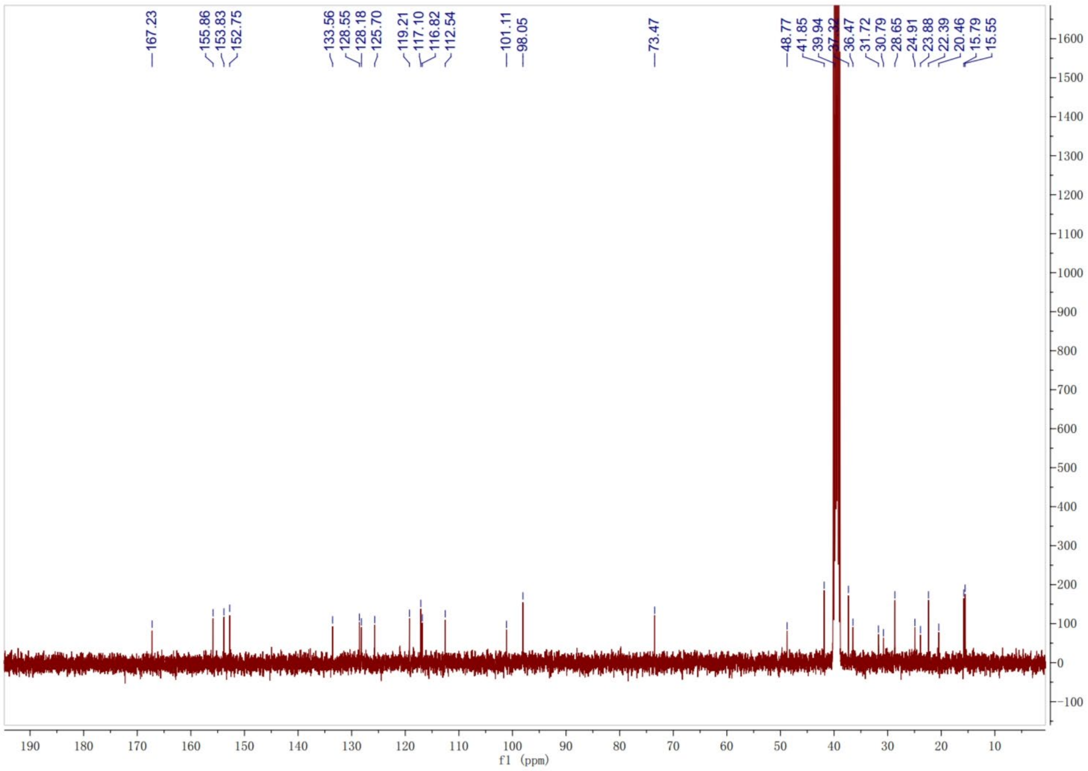

Figure S3 ^{13}C NMR Spectrum (100 MHz) of stachybotrin K (**1**) in DMSO-d_6

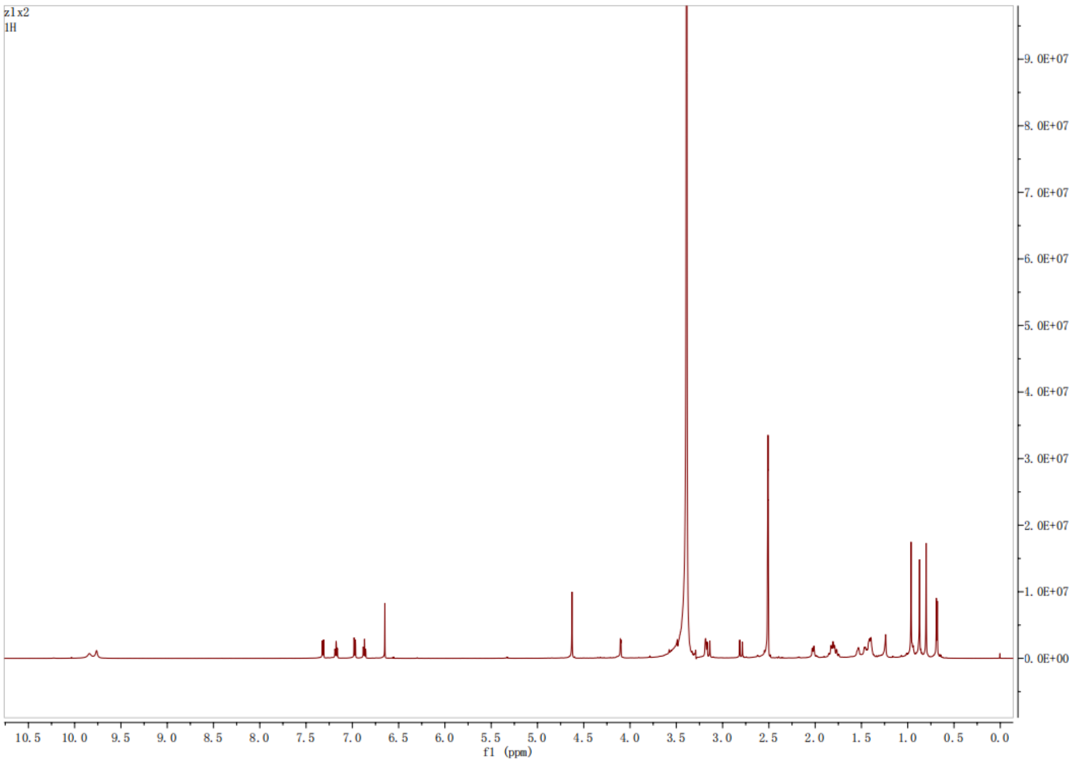

Figure S4 ^{1}H NMR Spectrum (600 MHz) of stachybotrin K (**1**) in DMSO-d_6

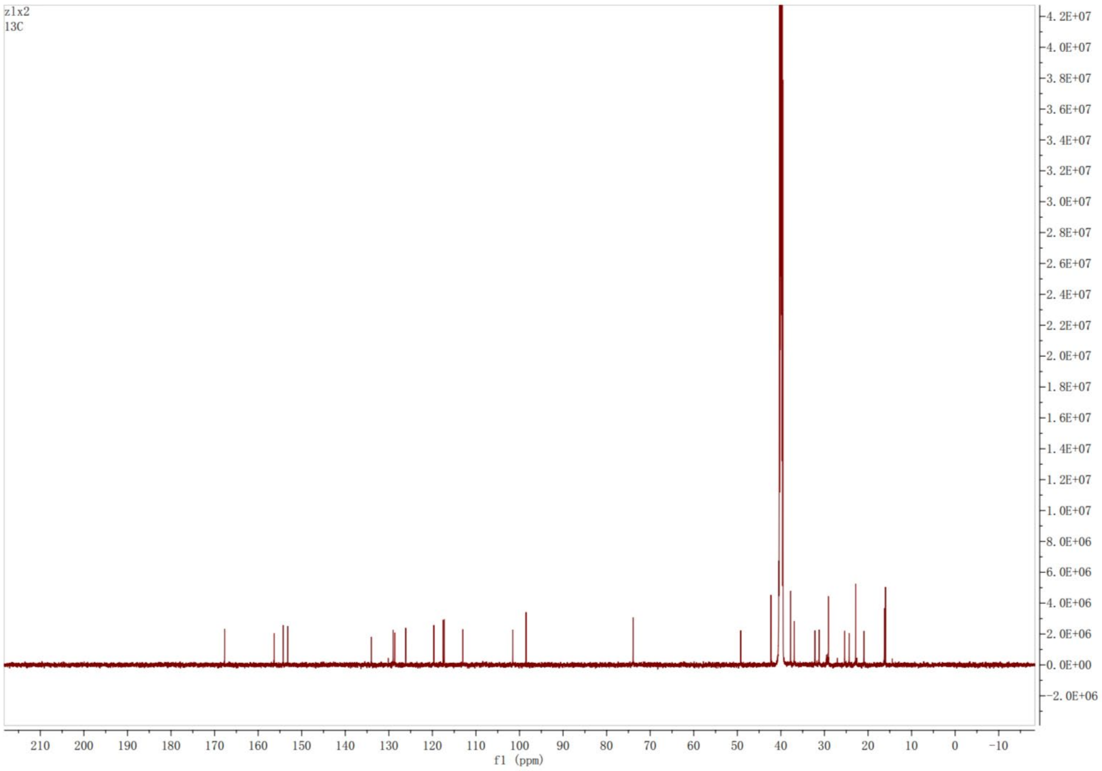

Figure S5 ^{13}C NMR Spectrum (150 MHz) of stachybotrin K (**1**) in DMSO-d_6

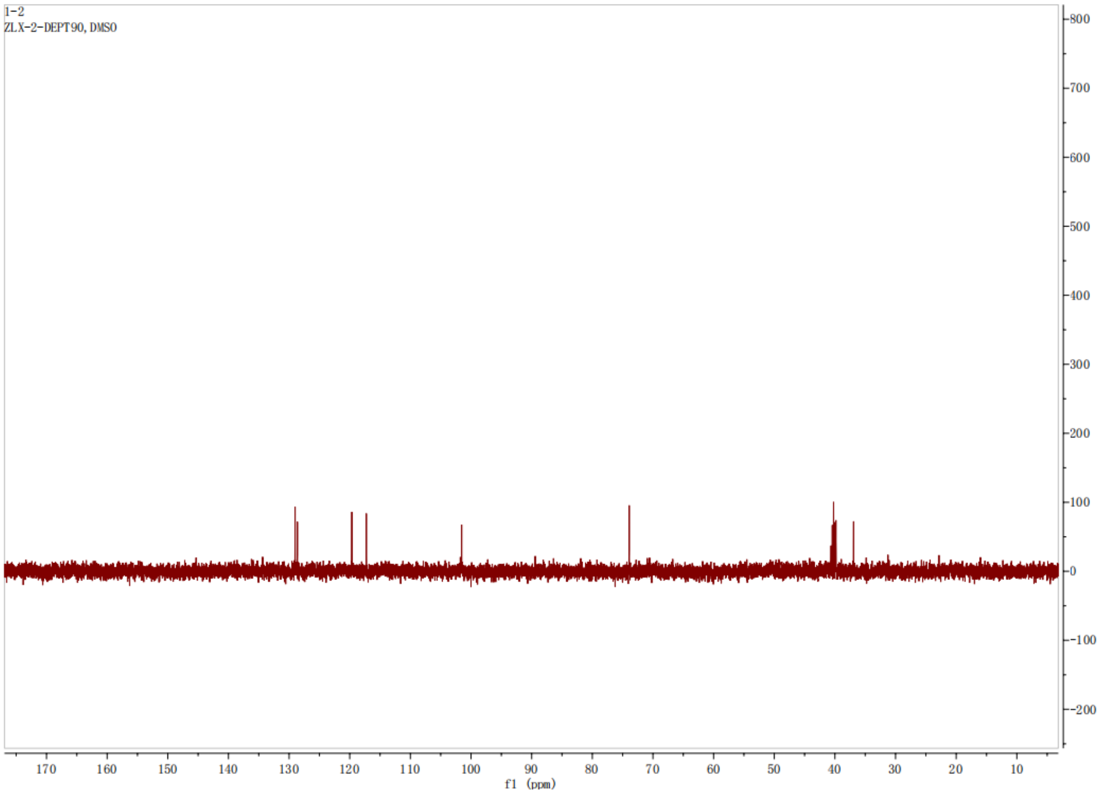

Figure S6 DEPT 90 Spectrum (100 MHz) of stachybotrin K (**1**) in DMSO-d_6

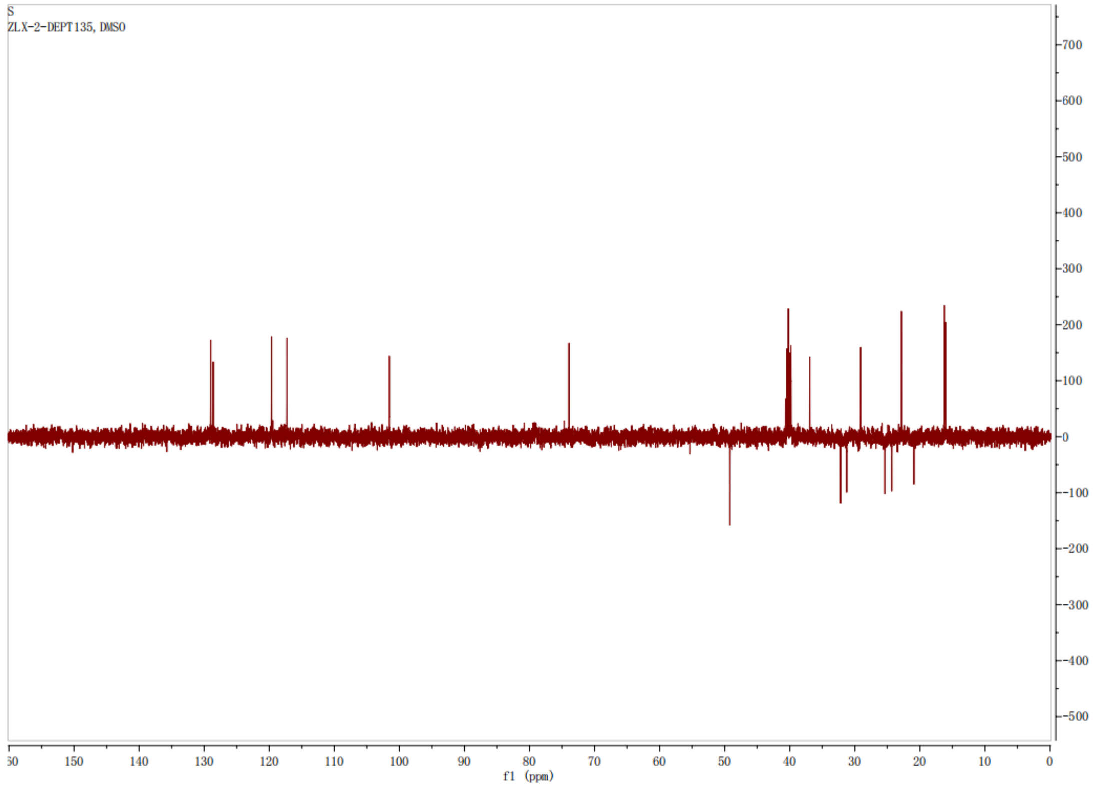

Figure S7 DEPT 135 Spectrum (100 MHz) of stachybotrin K (**1**) in DMSO-d_6

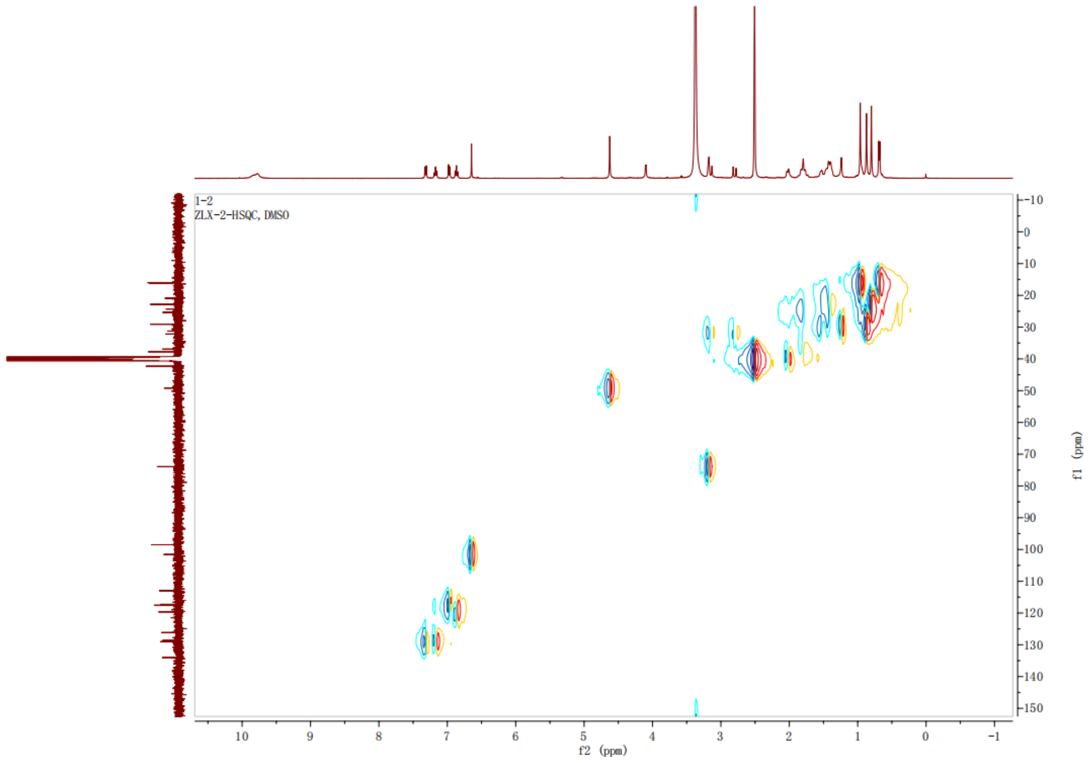

Figure S8 HMQC Spectrum of stachybotrin K (**1**) in DMSO-d_6

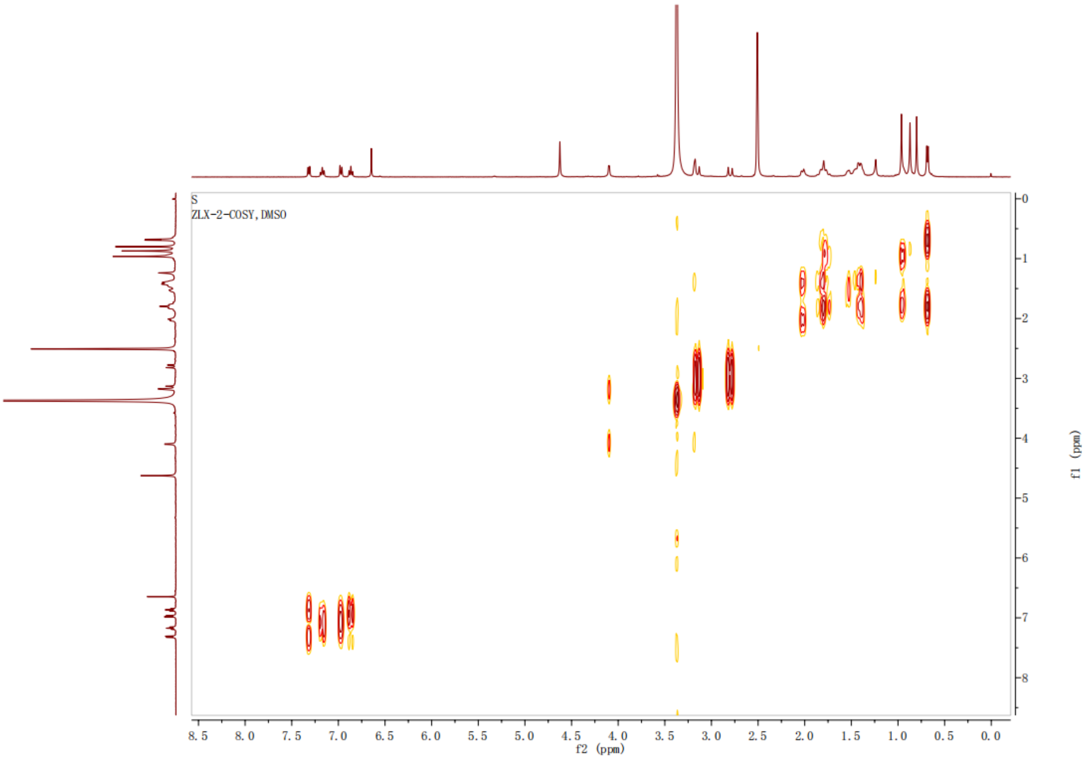

Figure S9 ^{1}H-^{1}H COSY Spectrum of stachybotrin K (**1**) in DMSO-d_6

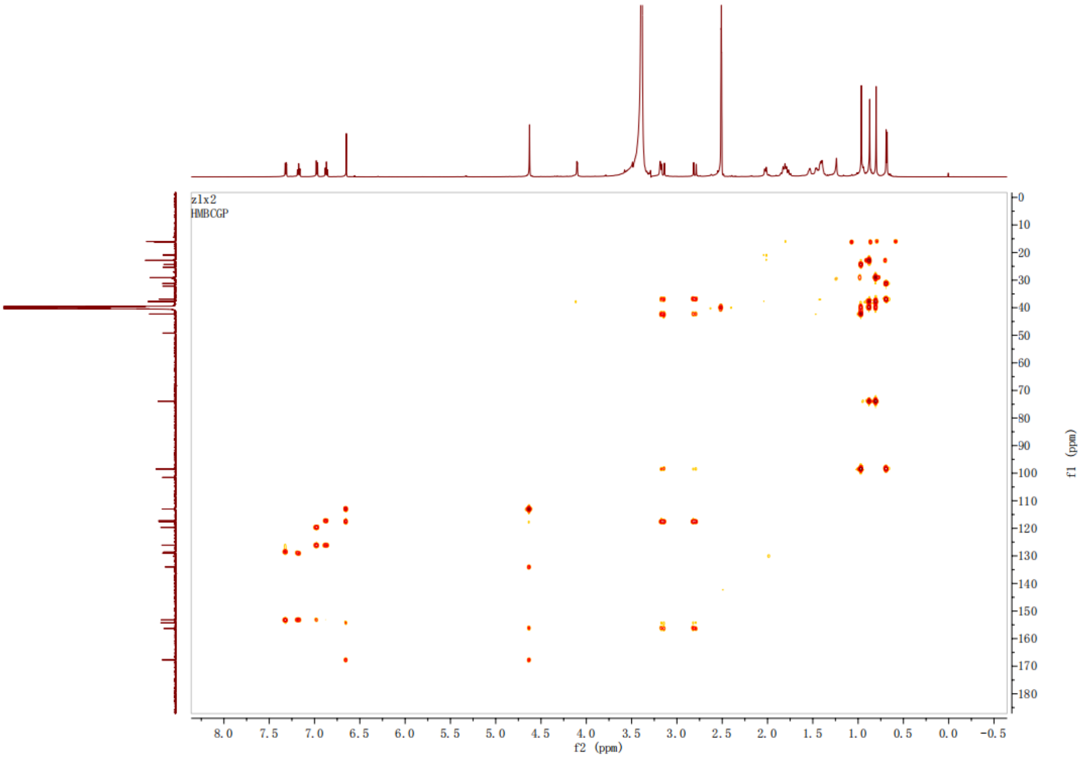

Figure S10 HMBC Spectrum of stachybotrin K (**1**) in DMSO-d_6

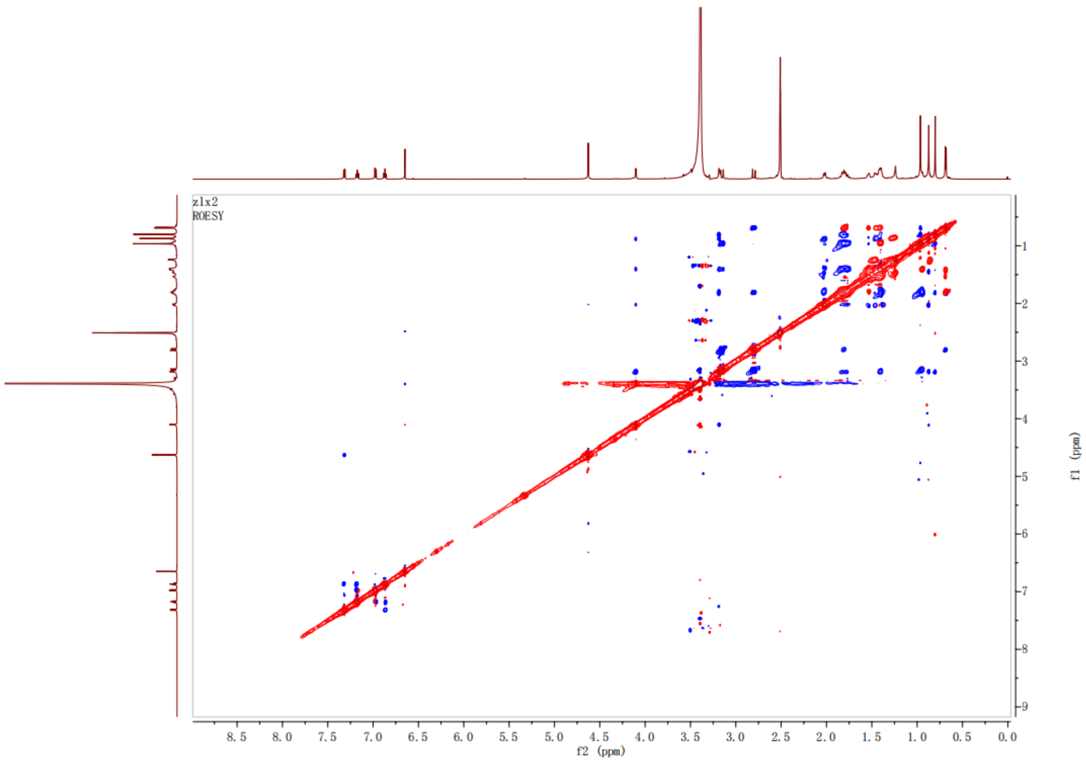

Figure S11 ROSEY Spectrum of stachybotrin K (**1**) in DMSO-d_6

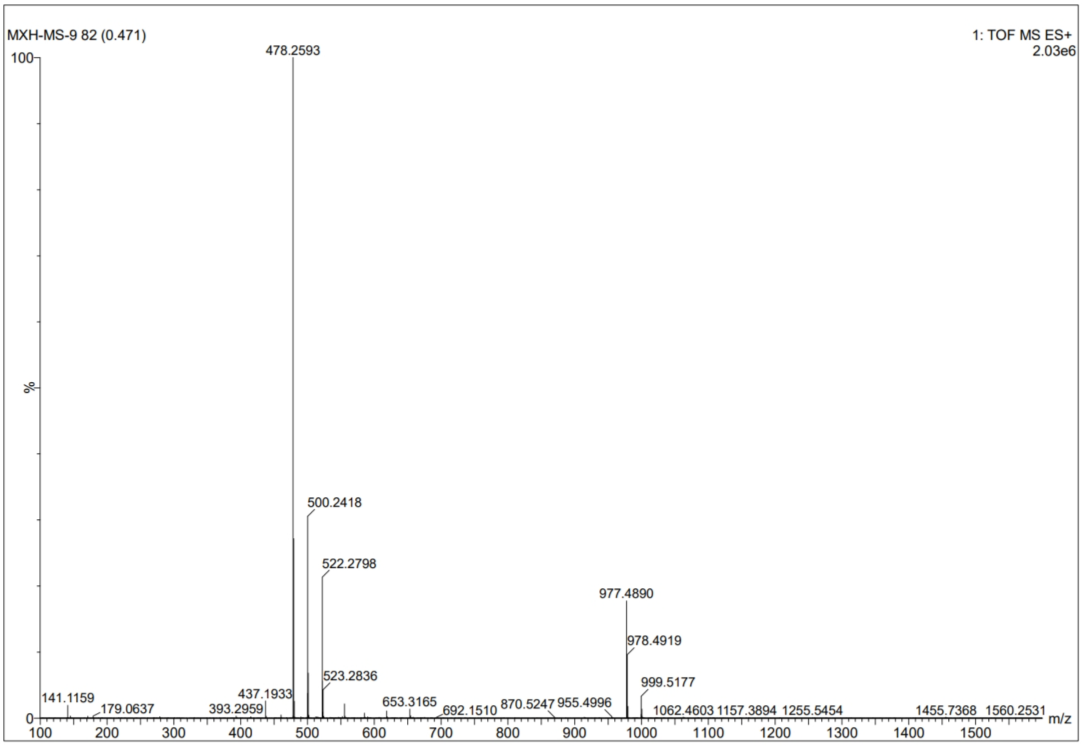

Figure S12 HRESIMS Spectrum of stachybotrin K (**1**)

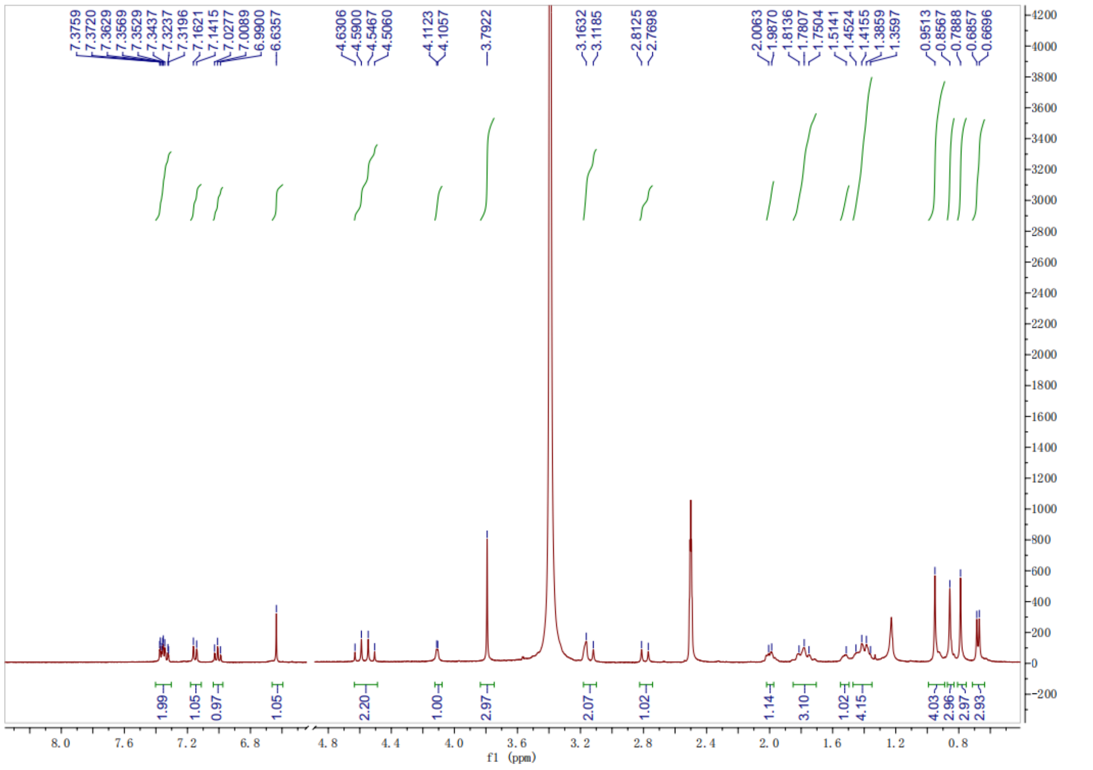

Figure S13 ^{1}H NMR Spectrum (400 MHz) of stachybotrin L (**2**) in DMSO-d_6

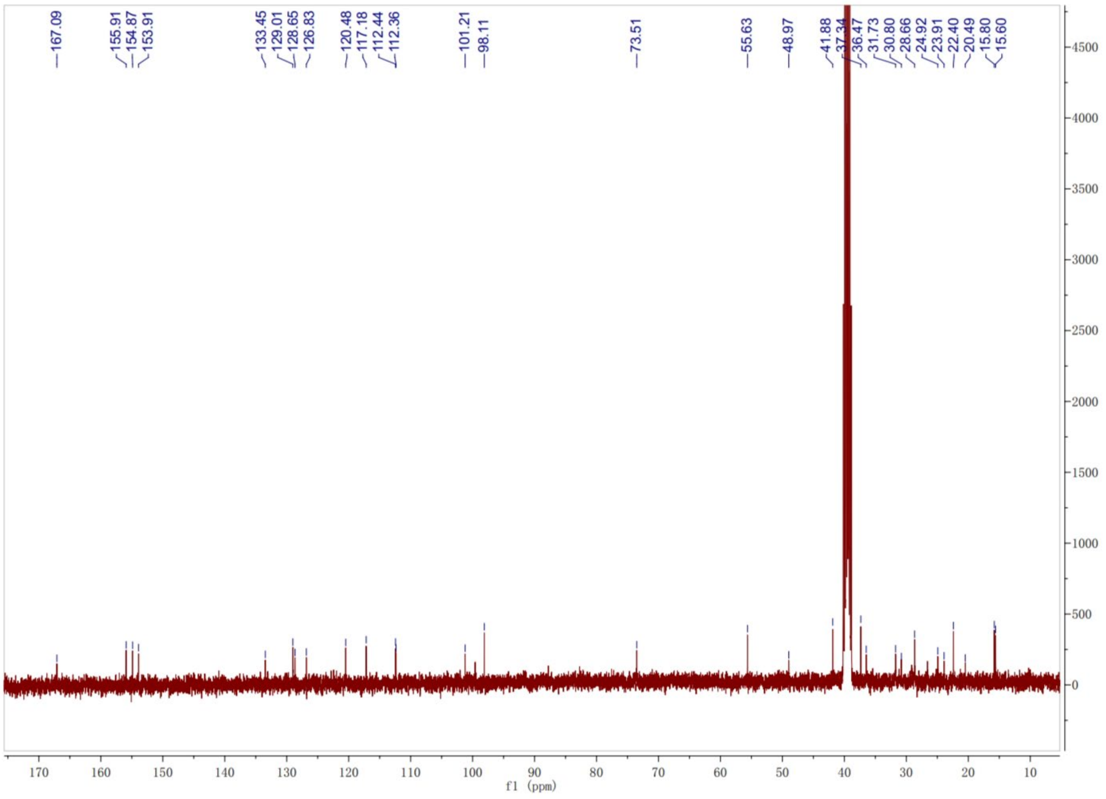

Figure S14 ^{13}C NMR Spectrum (100 MHz) of stachybotrin L (**2**) in DMSO-d_6

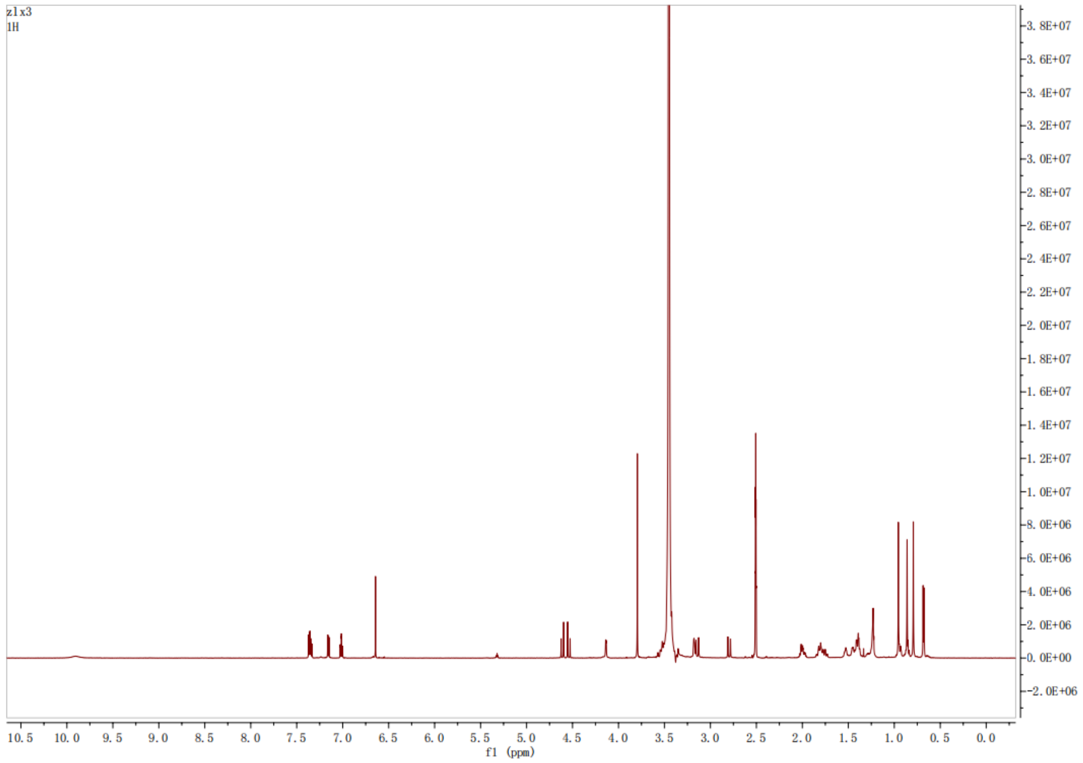

Figure S15 ^{1}H NMR Spectrum (600 MHz) of stachybotrin L (**2**) in DMSO-d_6

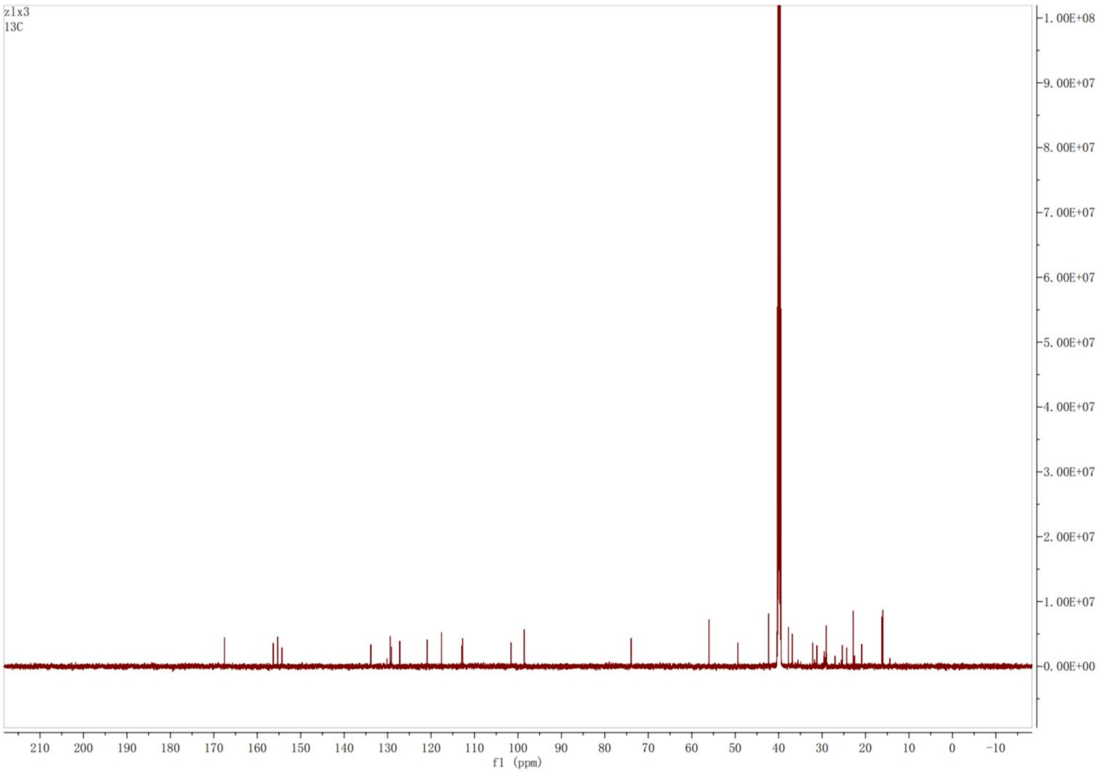

Figure S16 ^{13}C NMR Spectrum (150 MHz) of stachybotrin L (**2**) in DMSO-d_6

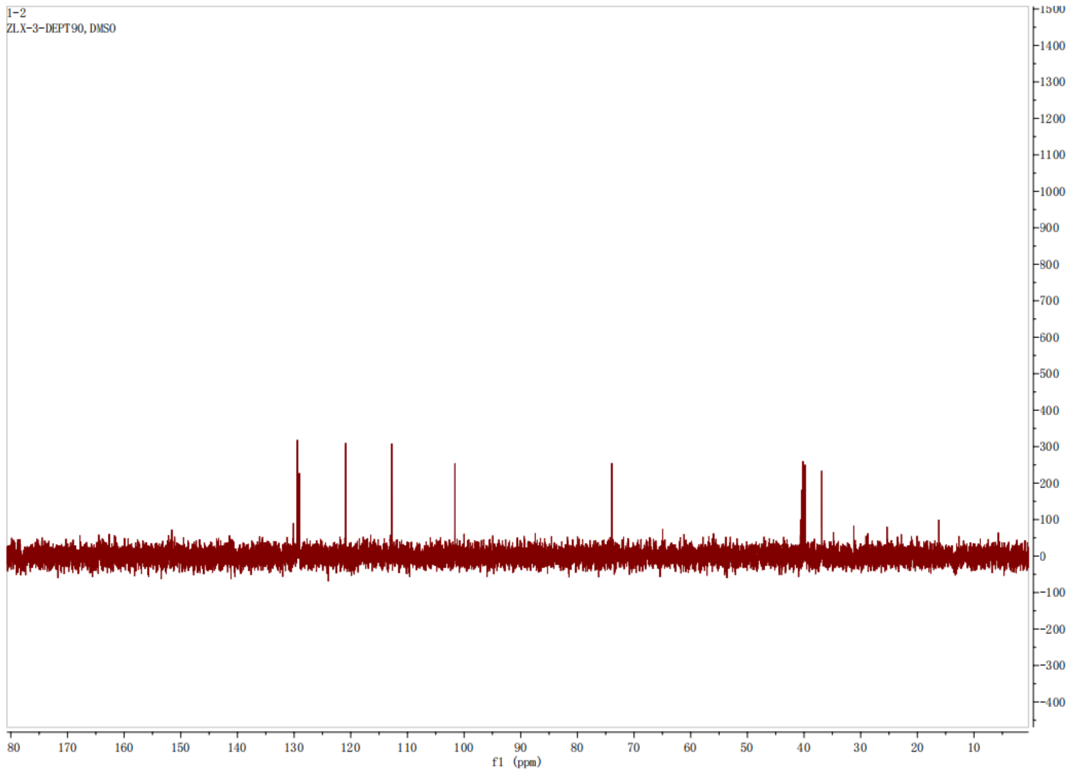

Figure S17 DEPT 90 Spectrum (100 MHz) of stachybotrin L (**2**) in DMSO-d_6

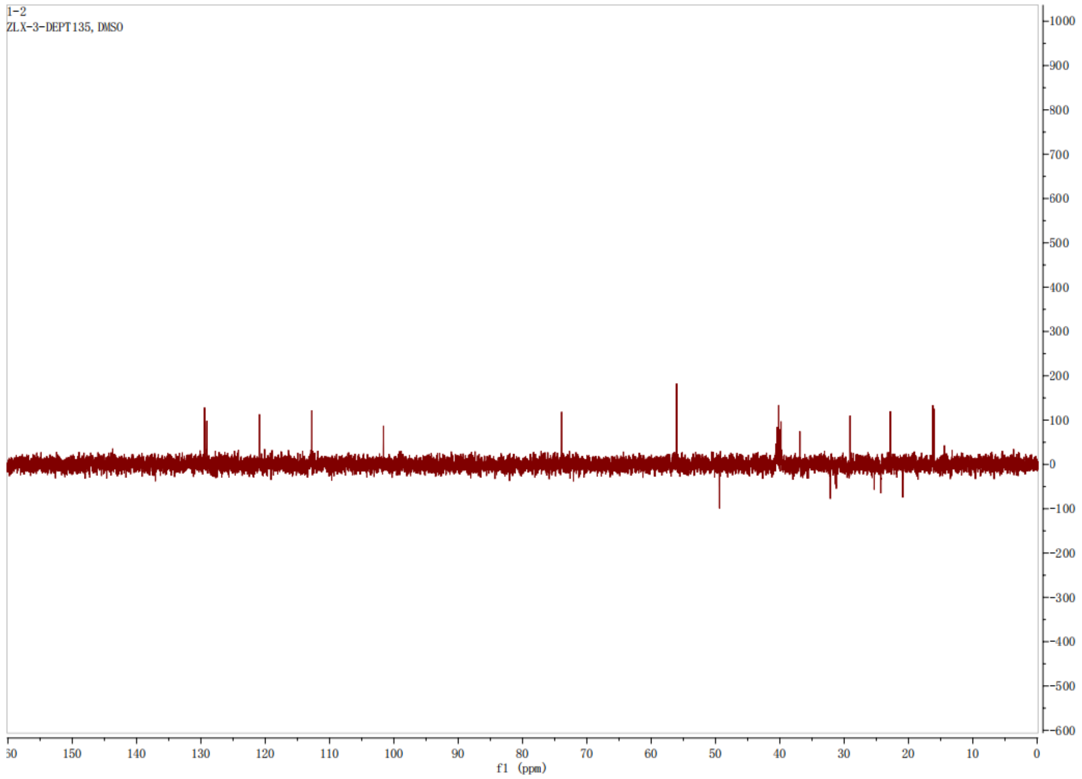

Figure S18 DEPT 135 Spectrum (100 MHz) of stachybotrin L (**2**) in DMSO-d_6

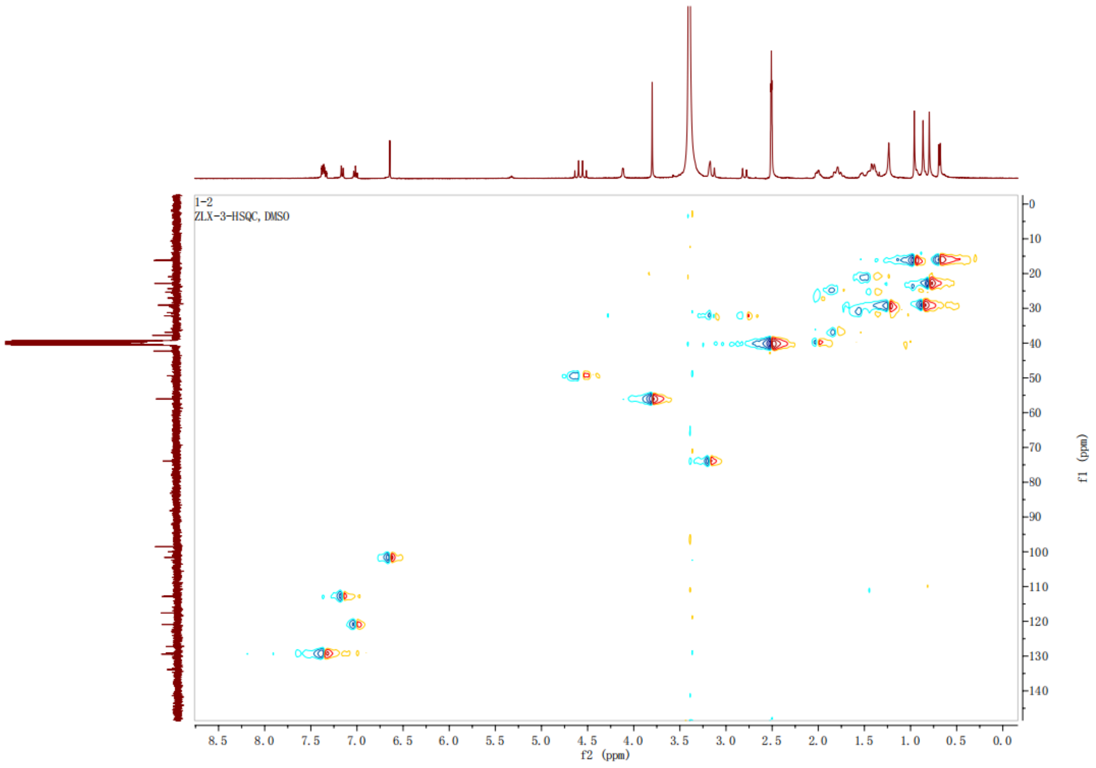

Figure S19 HMQC Spectrum of stachybotrin L (**2**) in DMSO-d_6

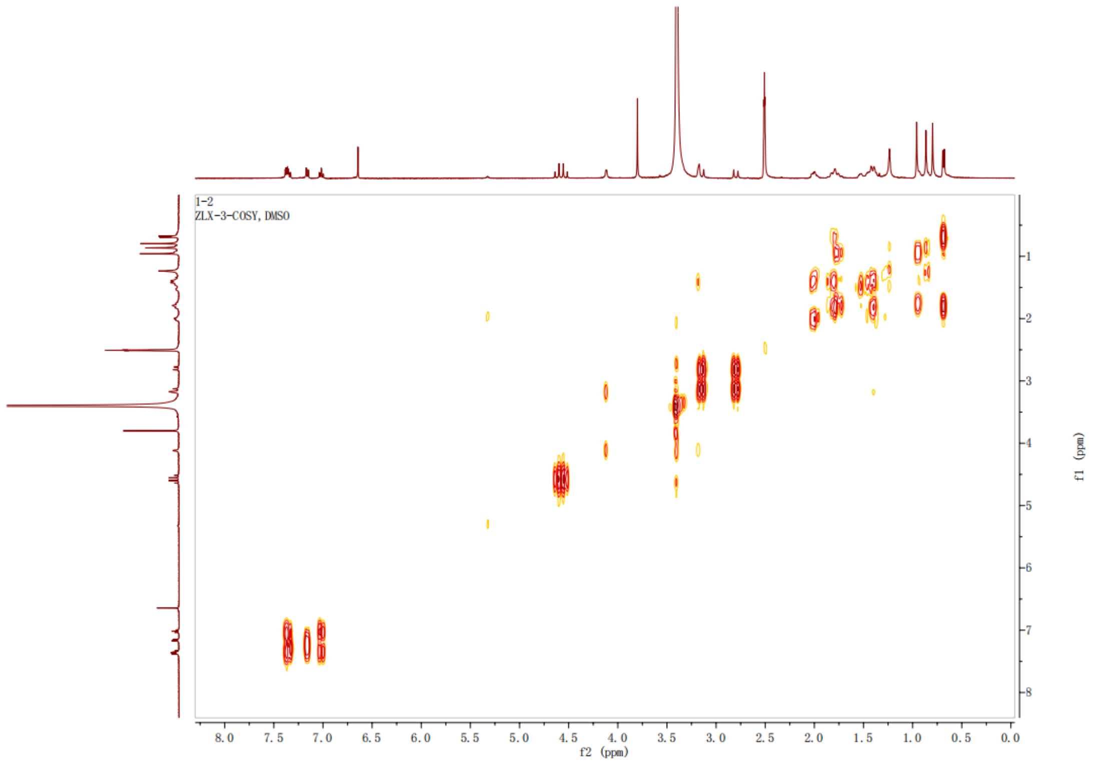

Figure S20 ^{1}H-^{1}H COSY Spectrum of stachybotrin L (**2**) in DMSO-d_6

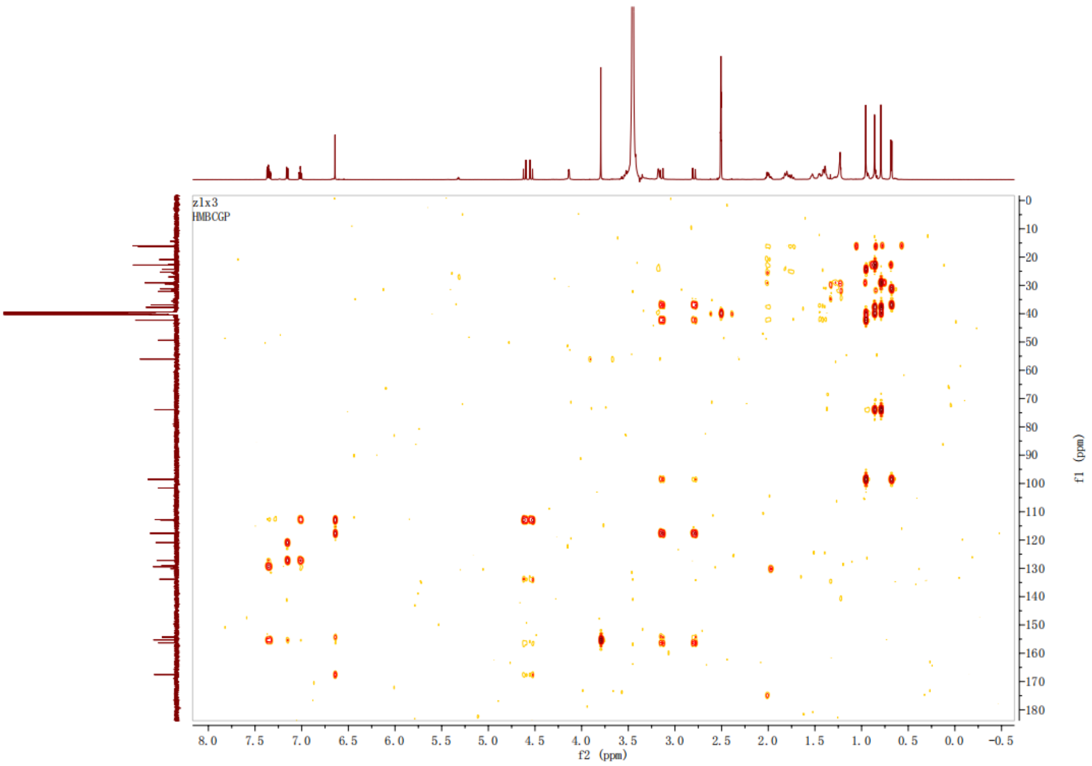

Figure S21 HMBC Spectrum of stachybotrin L (**2**) in DMSO-d_6

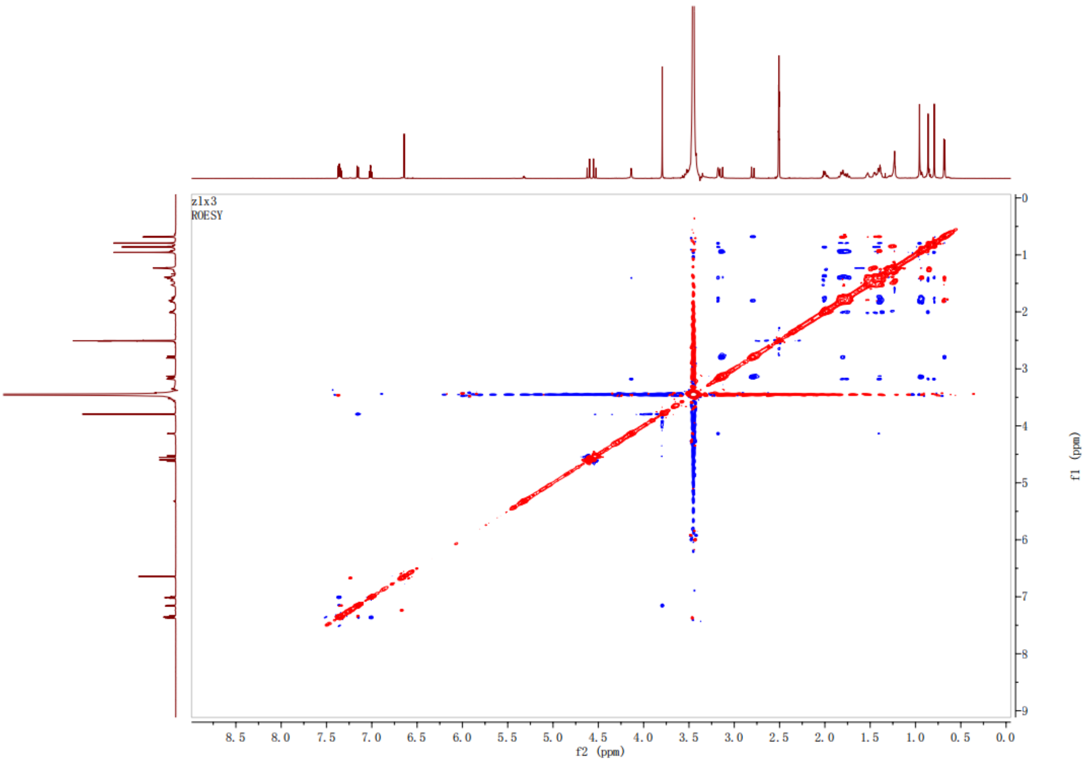

Figure S22 ROSEY Spectrum of stachybotrin L (**2**) in DMSO-d_6

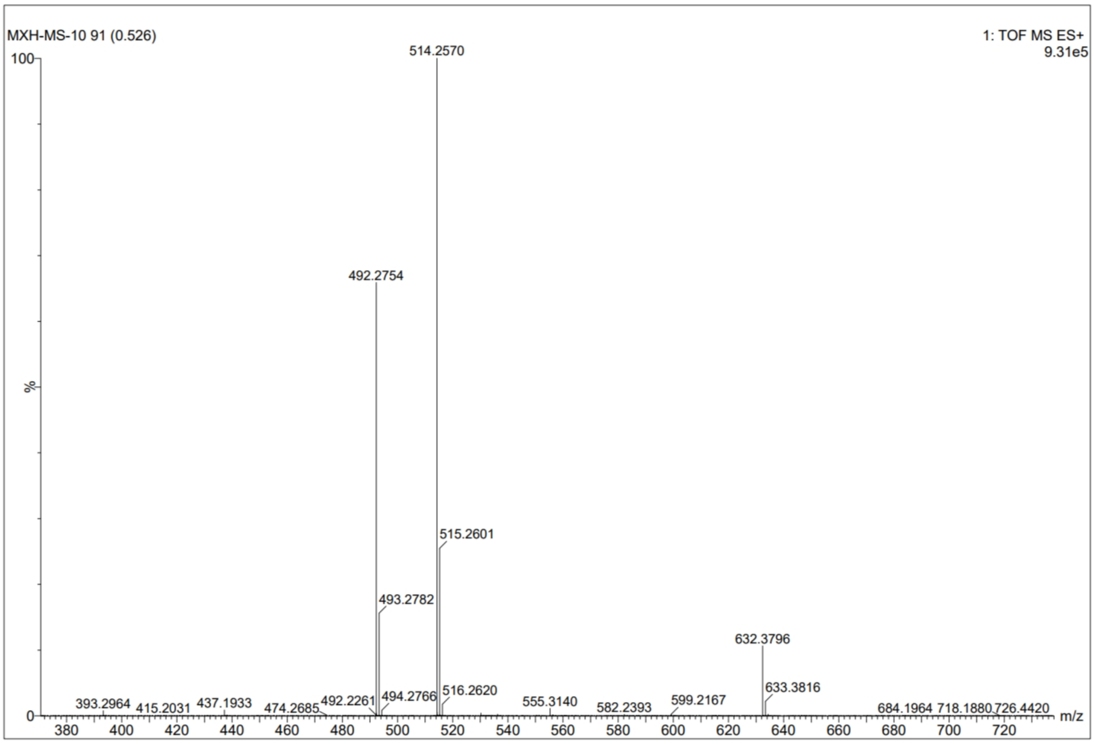

Figure S23 HRESIMS Spectrum of stachybotrin L (**2**)
